# Structure–Activity Relationship of Cyanine Dyes in Relation to the Hepatic Uptake and Excretion

**DOI:** 10.1002/cbic.202500762

**Published:** 2026-04-17

**Authors:** Nataliia S. Berehova, Emily L. J. de Bock, Maarten P. van Meerbeek, Chanel M. Naar, Thom van den Eng, Nikita A. Bouw, Tessa Buckle, Fijs W. B. van Leeuwen

**Affiliations:** ^1^ Interventional Molecular Imaging Laboratory (IMI) Leiden University Medical Center Leiden The Netherlands; ^2^ Leiden University Center for Infectious Diseases (LUCID) Leiden University Medical Center Leiden The Netherlands

**Keywords:** fluorescence imaging, fluorescent dye, hepatobiliary excretion, image‐guided surgery, liver surgery

## Abstract

**Background**: Indocyanine green (ICG) is a clinically approved fluorescent dye that is used for lesion identification during hepatobiliary surgery. However, the chemical structure of ICG seems to be suboptimal for this excretion‐based diagnostic readout. In this study the effect of systematic structural variations in Cy5‐analogs were evaluated to elucidate the structure–activity relationship between hepatic uptake and excretion.

**Methods**: Nine Cy5 dyes with variations in *N*‐alkyl indole substitution (butyl sulfonate or methyl), and benzoannulation were synthesized in analogy to the ICG scaffold (Cy7.5‐(SO3‐)2). Photo‐, bio‐ and chemical properties were analyzed and combined to identify structure–activity relationships using Spearman's correlation and multiparametric analysis.

**Results**: Chemical modifications were shown to alter (photo)chemical properties and constituted in clear biological effects; Sulfonate substitution supports hepatic excretion, while benzoannulation promoted nonspecific background accumulation. Cy5‐(SO3‐)2, rather than the ICG‐analog Cy5.5‐(SO3‐)2, was identified as lead.

**Conclusions**: Systematic evaluation revealed key structural determinants that influence biliary excretion and allowed lead selection of dyes for hepatobiliary imaging. These insights provide a foundation for the rational design of optimized fluorescent agents for this application.

## Graphical abstract

Systematic analysis of structural modifications on a Cyanine5‐dye backbone (in red; R_1/3_ = CH_3_, C_4_H_8_O_3_S^‐^; R_2/4_ =  H, SO_3_^‐^), their (photo)chemical properties and utility in the imaging of bile excretion in hepatocyte cultures. Multiparametric structure‐activity assessments indicate that positive charges drive mitochondrial retention, benzoannulation promotes nonspecific membrane staining, while sulfonate substitution supports the biologically relevant hepatic excretion.

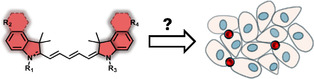

## 1 Introduction

Indocyanine green (ICG), a double‐sulfonated Cy7.5 dye, is one of the clinically most widely used fluorescent agents. Compared to other clinically applied dyes, such as fluorescein, ICG holds the unique property of being predominantly excreted via the hepatobiliary route [1]. This clearance profile has extended its clinical use during angiography [2], lymphangiography [3], and applications in hepatobiliary surgery [4, 5]. Here, the excretion of ICG in hepatocytes supports real‐time visualization of the biliary tree during cholecystectomy [6, 7, 8], identification of liver tumors [9, 10, 11], and surgical margins [12, 13, 14]. Uniquely, intrahepatic lesions are known to disrupt biliary clearance to the gall bladder, which results in local retention of ICG‐containing bile juice [15]. Depending on the lesion morphology, this yields either fluorescent rings or spheres that enable identification of the lesion during surgery [16].

ICG‐guided resection of liver metastasis was first introduced in 2009 (Japan) [9], has successfully been disseminated across the globe, and is still under active investigation [17]. A recent meta‐analysis by Xiong et al. has indicated that this procedure results in reduced blood loss, lower transfusion rates, shorter hospital stay, and lower complication rates [18]. At the same time, the same study noted that more clinical evidence is needed to evaluate the clinical value of this technique. To date, use of the ICG is driven by its low cost and medical approval, rather than its optimal structure–activity relationship for detecting liver metastases. One of the practical challenges encountered is that ICG also accumulates in unaffected liver tissue [5]. This unwanted side effect results in diminished the signal‐to‐background ratio's and thus the overall detectability of a lesion. Efforts to compensate for this effect have focused on tailoring the time between injection and surgery and decreasing the injected dose [19]. These modifications can help minimize the background intensity but do not overcome the intrinsic property of ICG to accumulate in hepatocytes. To prevent the latter, a dye that shows fast excretion and is not retained in hepatocytes is required.

Literature indicates that hepatobiliary surgery can be performed using a variety of chemical designs, including antibody–dye conjugates (e.g., SGM‐101 targeting CEA [20], M5A–IR800 [21], small‐molecule and peptide‐based probes (e.g., V‐1‐GGGK‐MPA targeting HGF [22], MPA–PEG4–Ang II targeting AGTR1 [23] and hybrid fluorescent–radioactive tracers (e.g., GB‐6 peptide [24]). While these tracers provide opportunities to improve tumor visualization, they are synthetically more complex to produce than ICG. Given the molecular simplicity of ICG (Cy7.5‐(SO_3_^‐^)_2_), one could argue that other synthetically straightforward Cy‐dye structures could accommodate a hepatic metabolism similar to or superior to that of ICG.

To investigate the structure–activity relationship between dye composition and hepatobiliary excretion a matrix of Cy‐dyes was designed and synthesized wherein the sulfonate and aromatic functionalization (benzannulation) on the Cy‐dye backbone were systematically varied. Herein the structural composition of ICG was used as a starting point. To ensure optimal compatibility with in vitro imaging modalities, a Cy5 rather than a Cy7 backbone was used. Next to (photo)chemical analysis, the uptake and excretion of the dyes within the matrix were evaluated in vitro. Both chemical and biological metrics were used to enable nonbiased selection of a lead compound that facilitates a fast clearance profile without accumulating in healthy hepatocytes.

## 2 Results

### 2.1 Synthesis and Matrix Composition

**FIGURE 1:**
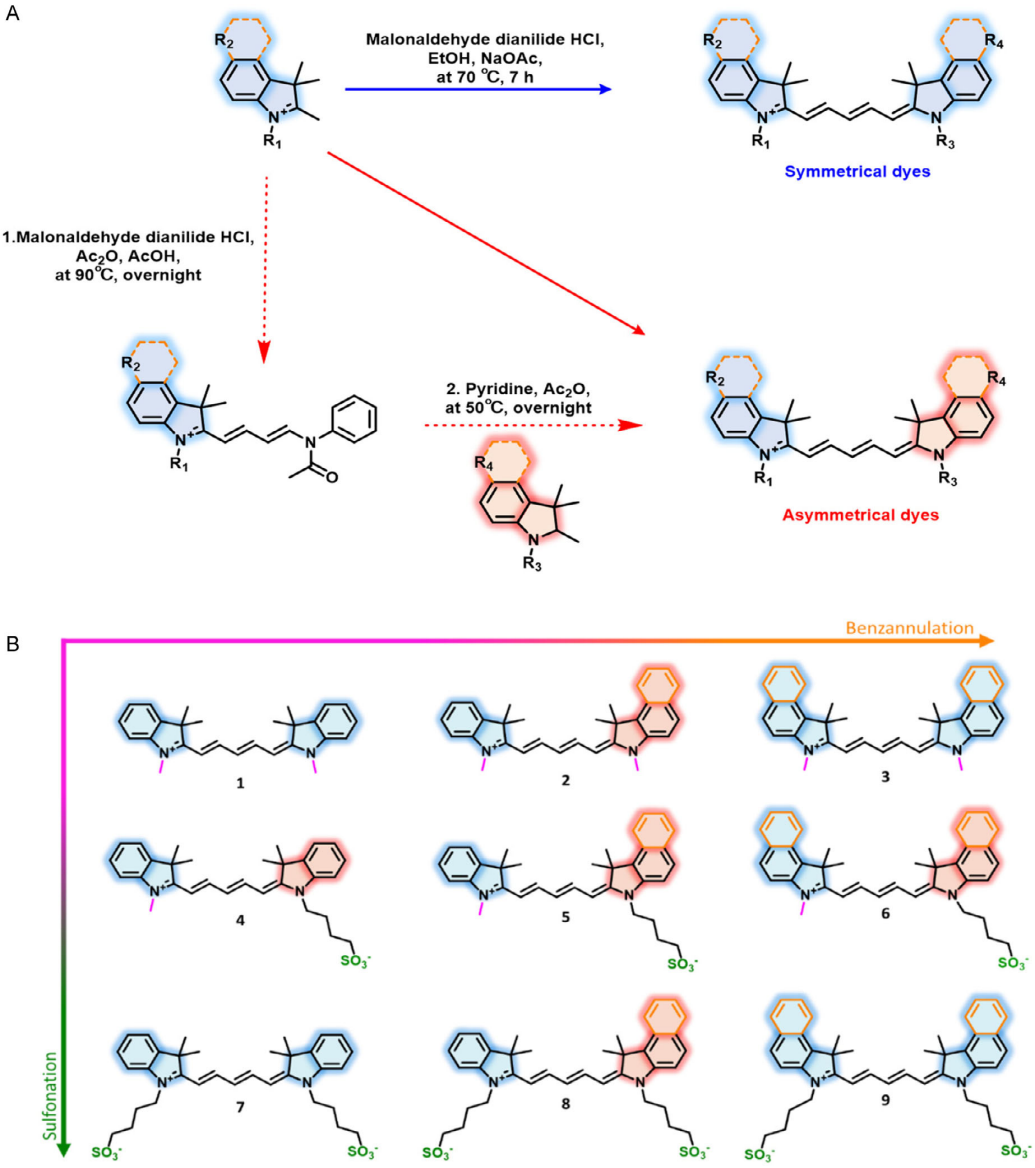
Synthesis and design of the Cy5 matrix. (A) Schematic visualization of the synthetic routes – Symmetrical dyes were obtained through a one‐step synthesis in EtOH under the governance of NaOAc. Asymmetrical dyes were obtained through a two‐step synthetic route by first forming the hemicyanine intermediate and then furnishing the dye by stirring in pyridine:Ac2O overnight; (B) Cy5‐core matrix design (R_1/3_ = CH_3_, C_4_H_8_O_3_S^‐^; R_2/4_ = H, SO_3_^‐^, Ar). Sulfonate groups are depicted in green. Sulfonation is shown from top to bottom; Benzene groups are depicted in orange. Benzannulation is shown from left to right; dye **1, 4,** and **7** contain no additional benzene group, dye **2, 5,** and **7** contain one sulfonate group and dye **3, 6,** and **9** contain two sulfonate groups.

Starting from the structural composition of ICG, a matrix of Cy‐dyes reengineered, wherein the sulfonate and aromatic functionalization (benzannulation) on the Cy‐dye backbone were systematically varied (Figure 1).

Two synthetic methods were employed to design the dye matrix (Figure 1A), yielding symmetrical and asymmetrical dyes through systematic variation of predefined building blocks. Specifically, the symmetrical dyes **1**, **3**, **7**, and **9** were synthesized via a one‐step condensation of two identical heterocyclic quaternary salts with a central polymethine linker, affording dark blue solids in relatively low yields (2.5%–30.6%).

The asymmetrical dyes **2**, **4**, **5**, **6**, and **8** were prepared by condensing a hetero‐pair of indolinium and benzothiazolium salts with the same polymethine linker, forming a hemicyanine intermediate that was subsequently converted into the final dyes in significantly higher yields (20.9%–70.2%). To further validate the enhanced efficiency of the asymmetric approach, dye **9** was resynthesized via a two‐step procedure, which increased the yield from 2.4% to 20.9%.

### 2.2 Chemical and Optical Properties

Table 1 summarizes the chemical and optical properties of the synthesized dyes. A systematic rise in lipophilicity logically followed the increase in degree of benzannulation (Figure 1B; left to right). Conversely, the introduction of sulfonate moieties reduced the lipophilicity (Figure 1B; top to bottom). No clear trend was observed for the serum binding; all compounds showed binding in the range of 70%–80%.

**TABLE 1:** Chemical and Optical properties of the synthesized cyanine dyes 1‐9.

Dye	Chemical properties		Optical properties	
	LogP_o/w_	Serum binding, %	Abs/Em, nm	Brightness
**1**	1.09 ± 0.03	81 ± 2	646/665	38 887
**2**	2.22 ± 0.06	80 ± 3	665/686	24 817
**3**	2.62 ± 0.05	78 ± 3	684/705	16 030
**4**	1.90 ± 0.07	71 ± 2	649/664	64 288
**5**	2.03 ± 0.18	81 ± 2	668/690	39 475
**6**	2.29 ± 0.11	75 ± 3	687/711	26 044
**7**	−1.73 ± 0.03	72 ± 2	652/677	54 649
**8**	−0.84 ± 0.02	73 ± 5	671/693	32 683
**9**	−0.10 ± 0.01	75 ± 4	688/710	31 280

In line with established knowledge, addition of every an extra benzene ring to the cyanine core resulted in a bathochromic shift of ≈20 nm (Table 1, Abs/Em), ranging from Cy5 (no additional ring; **1**, **4**, and **7**), to Cy5.25 (one additional ring; **2**, **5**, and **8**), and Cy5.5 (two additional rings; **3**, **6**, and **9**). Brightness followed similar trends – a reduction on brightness was seen following the introduction of aromatic moieties (Figure 1B left to right; Cy5 (**1**, **4**, and **7**) → Cy5.25 (**2**, **5**, and **8**) → Cy5.5 (**3**, **6**, and **9**)). An increase in the number of introduced sulfonate moieties resulted in altering dye charge (Figure 1B top to bottom; charge +1 (**1**‐**3**) → Charge 0 (**4–6**) → charge −1 (**7**‐**9**). For comparison the chemical and optical properties of ICG are provided in Table S1.

### 2.3 Cellular Uptake

**FIGURE 2:**
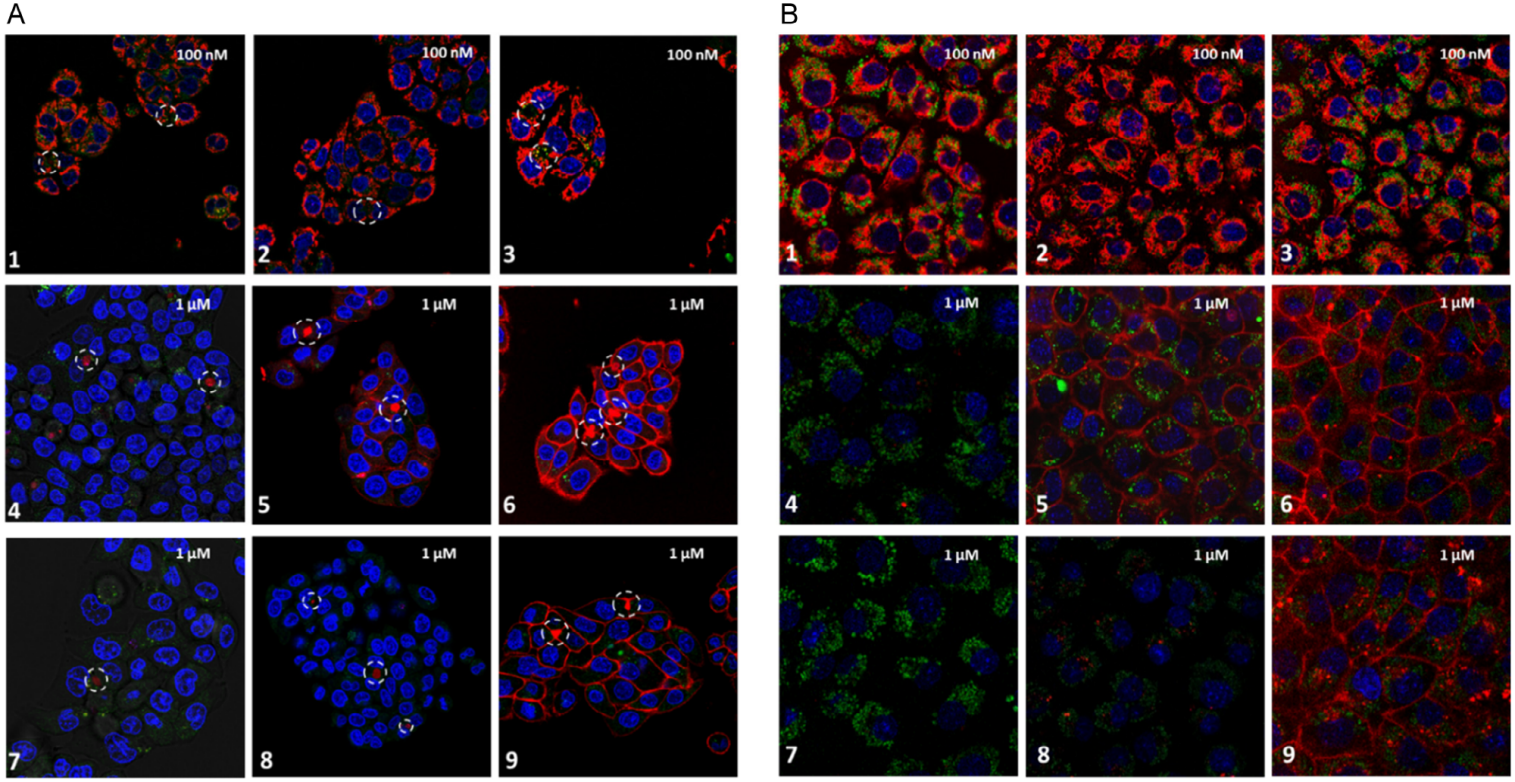
In vitro evaluation of dye uptake and excretion. Fluorescence confocal imaging of the Cy5‐dye matrix showing (A) Dye uptake in 2D Hepatocyte HC‐04.J7 cell line culture. Canaliculi are highlighted within a dashed white circle. (B) Dye uptake in epithelial Geβ3 control cells. Localization of the dye of interest in red, the cell nucleus in blue and lysosomes in green. Cells were incubated with either 100 nM (dye **1–3**) or 1 μM (dye **4–9**).

**FIGURE 3:**
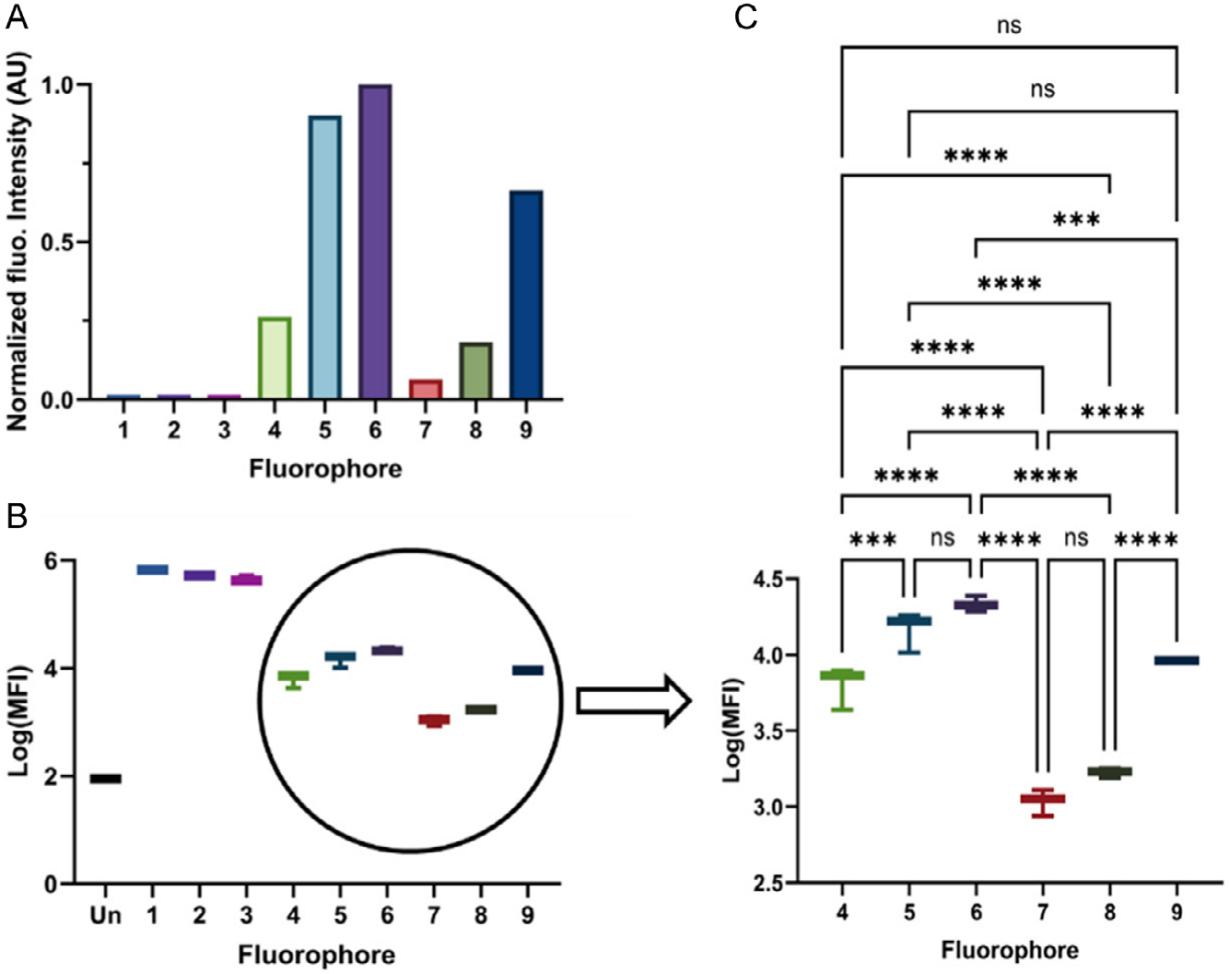
Uptake in hepatocytes. (A) Qualitative canaliculi uptake measurement of dye **1**‐**9** (derived from confocal microscopy images; average of *N* = 10). (B) Quantitative cellular uptake measurement of the dyes **1**‐**9** (collected using flow cytometry; average of *N* = 3 per dye). (C) Statistical comparison of cellular uptake in **4**‐**9** based on flow cytometry data**.** ns = nonsignificant, ***: *p* = 0.0002, ****: *p* = 0.0001.

To evaluate the uptake and excretion of compounds **1–9,** uptake patterns in hepatocytes were assessed in a single‐layer hepatocyte cell culture (HC‐04.J7 cell line with OATP1B1 transporter expression, Figure S28). Fluorescence confocal microscopy showed that positively charged dyes with a double methyl substitution showed consistent mitochondrial accumulation (Figure 2A, **1–3**), without seeing clear influences following benzannulation. Substitution of the cyanine core with a negatively charged tail shifted dye uptake to canaliculi located between hepatocytes (Figure 2A, **4**‐**9;** canaliculi accentuated by dashed circle and Figure 3A). According to literature, such localization in canaliculi suggests metabolism and excretion via bile [25]. Notably, only dyes **4**, **7**, and **8** showed exclusive canaliculi accumulation, while for dyes **5**, **6**, and **9,** it was combined with membrane staining. The latter indicates that benzannulation drives membrane staining. Interestingly, in **8,** the double sulfonation seems to counterbalance the introduction of the benzene group. Results obtained with (ICG) (bis‐sulfonated Cy7.5 analog), show canaliculi uptake combined with a membrane staining (Figure S29).

To further confirm the effect of dye substituents on bile excretion control experiments were performed in an epithelial cell line (Geβ3 cells, (Figure 2B), a cell line that expresses an unglycosylated form of the OATP1B1 transporter [26] (Figure S28)). In line with the results obtained in hepatocytes mitochondrial staining was observed for dye **1–3** and no intracellular Cy5 uptake was observed for **4**, **7**, and **8** in the control cells. Bile excretion was not observed due to the absence of bile canaliculi in the epithelial cells. Dyes **5**, **6**, and **9** again showed uptake in a membrane (Figure 2B).

Retention of the dyes in the cells was quantitatively determined via flow cytometry (Figure 3B). In line with mitochondrial staining seen on confocal imaging, dyes **1**–**3** demonstrated a high fluorescence signal with similar intensities. When focusing on the dyes that displayed transport to canaliculi on confocal, higher uptake values were found for the dyes that also stained the membrane (**5**, **6,** and **9**). Dyes **4**, **7,** and **8** showed significantly lower cell retention (Figure 3C). The latter suggests fast dye transport from the cell to the cannula. This comparison underscores that sulfonation reduces nonspecific uptake, while benzannulation promotes it.

No toxicity was observed for any of the dyes in any of the conditions investigated.

### 2.4 Selection of a Lead Structure

**FIGURE 4:**
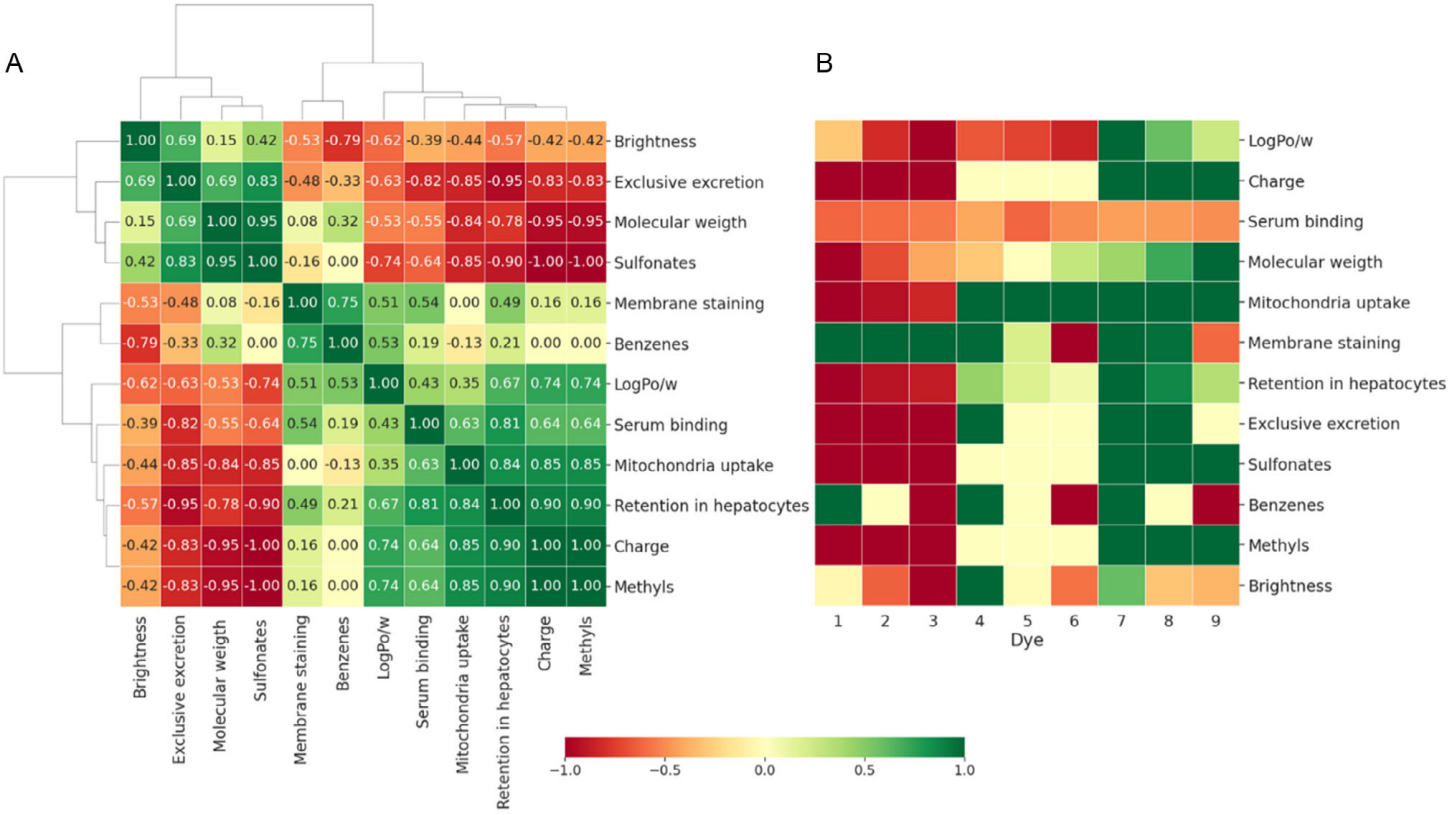
Structure‐activity relationship of dye features. (A) Clustered Spearman correlation heatmap. Color bar was set from green (+1) to red (−1), where +1 (perfect monotonic relationship) to −1 (perfect negative monotonic relationship), and 0 indicating no monotonic relationship. (B) Multiparametric feature evaluation. The color map from −1 to +1 was used, where −1 (red) represents an unfavorable influence and +1 a favorable influence on exclusive hepatobiliary excretion.

To determine the structure–activity relationship that promotes hepatobiliary clearance, Spearman correlation analysis was performed (Figure 4A). This was further combined with hierarchical clustering on chemical composition (benzene, methyl, and sulfonate substitution, molecular weight and charge), chemical properties (LogP and serum binding) of the dyes, their cellular accumulation (membrane and mitochondrial staining, and retention in hepatocytes), and their hepatobiliary excretion (exclusive excretion). Metrics in proximity within the clustering analysis exhibited stronger correlations. Sulfonate substitution was shown to demonstrate a strong positive correlation with biliary excretion of the dyes (*r* = 0.83; in green). Increased molecular weight had a favorable effect on the latter (*r* = 0.69; in green).

In contrast, methyl substitution and *a* + 1 charge were associated with a strong negative correlation (*r *= –0.83; in red). No strong correlation was observed for benzannulation (*r* = −0.33). However, the negative value indicates an unfavorable influence of additional benzene rings on hepatobiliary excretion. The same trend was seen for chemical properties, where higher percentages of serum binding and higher lipophilicity values showed strong negative correlations (*r* = −0.82 and *r* = −0.63, respectively). Consistent with previous findings, high levels of dye retention were strongly linked to mitochondrial uptake (*r* = −0.85). Higher numbers of membrane staining also favored dye retention in cells without providing a strong correlation.

Based on Spearman's correlation a multiparametric assessment was performed (Figure 4B). The latter represents the positive (green) or negative (red) effects of each feature on the biliary excretion of the dye and allows for unbiased selection of the optimal dye. The highest number of green outcomes in this assessment, and therefore the most favorable characteristics could be contributed to dye **7**. As such, this dye was selected as the most optimal.

## 3 Discussion

Analysis of photo‐, bio‐ and chemical properties of nine structurally different Cy‐5 dyes (Figure 1B) allowed evaluation of the structure–activity relationship that drives hepatobiliary clearance of fluorescent dyes suitable for use in hepatobiliary surgery.

The presented results indicate that uptake of lipophilic and positively charged dyes (**1**‐**3**) is driven by the membrane potential [27], accumulate in mitochondria, and are not excreted (Figures 2, 3). From the literature, it is known that uptake of ICG (charge −1 and 2 ‐SO_3_^‐^ moieties) in hepatocytes is primarily regulated via the OATP1B1 transporter [28, 29, 30, 31]. Consistent with this, only sulfonated dyes (**4**–**9**) from the matrix show excretion in the OATP1B1 positive hepatocytes (Figure 2A, Figure S28), but not the OATP1B1 negative epithelial control cells (Figure 2B, Figure S28). Due to their higher LogP values dyes **5**, **6**, and **9** (the latter being a Cy5 analog of ICG) demonstrate prominent nonspecific membrane uptake, which also results in background staining (Table 1, Figures 2, and 3). Combined, chemical modifications in the dye structure could be coupled to clear biological effects (Figures 2‐4), which resulted in the selection of a lead compound (dye **7**, Cy5‐(SO3)2) that showed hepatic clearance without nonspecific background accumulation.

In this study, we focused on the Cy5 backbone as its emission in the 660–710 nm range is highly compatible with widely accessible analytical techniques such as confocal microscopy and flow cytometry. Compared to the Cy7 backbone of ICG, Cy5 lacks two carbon atoms in the polymethine bridge, resulting in a slightly lower LogP (Table S1). While such structural changes are known to affect pharmacokinetics in vivo [32], the bis‐sulfonated Cy5.5 analog produced in vitro results similar to those of its bis‐sulfonated Cy7.5 analog, for example, canaliculi uptake combined with a membrane staining (ICG; Figure S29). Along with recently published *h*HEPATO data [25], which also used a Cy5 backbone, these findings indicate that Cy5 analogs are equally suitable for hepatobiliary imaging. Important to note is that Cy5 has already been successfully implemented in several image‐guided surgery trials [33, 34]. Future studies are needed to determine whether the findings presented here directly translate to a Cy7 backbone.

While we recognize that the in vitro mechanisms investigated in this study are directly related to the in vivo accumulation of dyes in hepatic lesions [25], our primary objective was not to introduce a new chemical entity for immediate clinical translation. Instead, we aimed to provide mechanistic insight into the structure–activity relationships underlying the behavior of Cy‐dyes in hepatobiliary image‐guided surgery. Nevertheless, the design of compound 7 supports scalable GMP production, potentially allowing future manufacturing costs to approach those of ICG (≈90 euros per 25 mg vial). Clinically, an optimized tracer design could offer significant advantages by streamlining intraoperative workflows and minimizing hepatic background retention.

## 4 Conclusions

Through a systematic analysis of structural modifications on a Cy5‐dye backbone, this study has identified the core properties that define hepatic excretion in its simplest form. Uniquely, clustered Spearman correlation and multiparametric feature evaluation allowed alignment of (photo)chemical properties with desirable biological behavior. Specifically, the structure–activity relationship analysis indicates that sulfonate substitution of dyes tends to support hepatic excretion, while benzoannulation of the same dye backbone promotes nonspecific uptake. As a result, Cy5‐(SO_3‐_)_2_ was identified as lead, rather than Cy5.5‐(SO_3‐_)_2_ which functions as analog of Cy7.5‐(SO_3‐_)_2_ (also called ICG). The fact that the chemical structure of the identified lead differs from that of ICG indicates that image‐guided hepatobiliary surgery may benefit from further chemical innovations. Moreover, our structure–property evaluation strategy provides a general framework applicable to other biomedical contexts where precise tuning of chemical properties is needed to achieve specific biological or diagnostic outcomes.

## 5 Experimental

### 5.1 Chemistry

All chemicals were procured from Actu‐All Chemical (Oss, The Netherlands), Sigma Aldrich (St. Louis, MO, US), Tokyo Chemical Industry (Tokyo, Japan), Biosolve BV (Valkenswaard, The Netherlands), and VWR Chemicals (Solon, OH, US) and used without additional purification unless otherwise specified. Dimethyl sulfoxide (DMSO) and Dimethylformamide (DMF) were desiccated over 4 Å molecular sieves for a minimum of 24 h before use.

To get to the matrix of 9 Cy5 dyes, different synthetic building blocks had to be synthesized from commercial products, following previously published procedures [34, 35, 36, 37]. Final symmetrical and asymmetrical dyes were synthesized following two different synthetic routes (Figure 1).

For reversed‐phase purification, preparative high‐pressure liquid chromatography (prep‐HPLC) was used with a Waters HPLC system (Waters Chromatography B.V., Etten‐Leur, The Netherlands), comprising a 2545 quaternary gradient module pump and a 2489 UV detector. Mass analysis was performed on a Waters Acquity system using a UPLC photodiode array detector, an SQ Detector mass spectrometer, and a flow rate of 0.5 mL/min (Waters [EH C18 130 Å 1.7 mm (100 x 2.1 mm) column). ^1^H‐NMR spectra were recorded on an AV‐400‐WB or AV‐600 high‐performance nuclear magnetic resonance (NMR) spectrometer (Bruker, Billerica, MA, USA). Detailed general procedures, ^1^H‐NMR, ^13^C‐NMR, and mass characterization of the Cy5 dyes **1**‐**9** id reported in the Supporting Information (Figures S1‐27).

### 5.2 Partition Coefficient (LogP)

The compound's partition coefficient (LogP) between 1‐octanol and water was determined using a modified shake flask method. The absorbance intensity of separated phases was measured using a UV1280 UV–vis spectrometer (Shimadzu, Kyoto, Japan), and concentrations were calculated using the Beer–Lambert Law. The LogP value was then calculated as the logarithm of the ratio of compound concentration in 1‐octanol and water.

### 5.3 Serum Protein Binding

The compound's serum protein binding was determined according to a previously reported procedure based on equilibrium dialysis [38]. In brief, heat‐inactivated Fetal Bovine Serum (FBS) was added to the dialysis chamber of a single‐use Rapid Equilibrium Dialysis (RED) plate (Thermo Fisher Scientific, Waltham, USA), followed by the addition of 3 μL of a 100 μM dye. Phosphate‐buffered saline (PBS) was added to the reservoir chamber, and the plate was incubated overnight at room temperature with swirling. Aliquots (100 μL) from both chambers were measured for fluorescence using a Perkin‐Elmer LS55 using a white 96‐well plate (Greiner Bio‐One). Serum protein binding was calculated as

$${\%}_{\text{bound}}=\left(100-\frac{\left[\text{buffer}\;\text{chamber}\right]}{\left[\text{serum}\;\text{chamber}\right]}\right)\cdot 100$$

### 5.4 Photophysical Properties

Absorbance and fluorescence measurements were carried out with the UV1280 UV–Vis spectrometer (Shimadzu, Kyoto, Japan) and LS55 instrument (Perkin Elmer, Waltham, MA, US), respectively. Molar extinction coefficients (*ε*) were determined in DMSO. Relative quantum yield (Φ_F_) determination was performed as previously described [38]. Dyes 1 (Φ_F_ = 37%), 2 (Φ_F_ = 23%), and 3 (Φ_F_ = 14%) were used as a reference dye for the Cy5, Cy5.25, and Cy5.5 groups, respectively. The brightness of the fluorophores was calculated by multiplying the corresponding molar extinction coefficient by the corresponding relative quantum yield.

### 5.5 In Vitro Analysis

In line with previous work [25], the in vitro evaluation of the hepatobiliary metabolism of fluorophores was carried out on immortalized hepatocyte cells (HC‐04.J7). Experiments in epithelial cells (Ge*β*3), conducted under identical conditions, were used for control purposes. Both cell lines were cultured in Dulbecco's modified Eagle medium (DMEM) (Life Technologies, Paisley, UK) containing penicillin, streptomycin, and fetal calf serum (FCS, 10%) (all BD Biosciences, Franklin Lakes, New Jersey, US) at 37°C and 5% CO_2_.

Cells were seeded onto glass‐bottom confocal dishes as previously described [25]. Prior to fluorescence confocal microscopy imaging, cells were incubated with LysoTracker Green DND‐26 (2 µL/mL, Invitrogen, Waltham, Massachusetts, US) and Hoechst 33 342 (33 342, 5 µL/mL; Invitrogen, Waltham, Massachusetts, US) for 30 min at 37°C, followed by rinsing with phosphate‐buffered saline (PBS). Subsequently, samples were incubated with 1 µM of cyanine‐dye solution (**4–9**) in DMEM for 10 min. For **1–3** the dye concentration was reduced to 100 nM to avoid oversaturation of the signal.

After incubation, samples were rinsed three times with PBS and imaged immediately using a Leica SP8‐WLL confocal microscope (63x magnification, Leica Microsystems GmbH, Wetzlar, Germany) using transmission and fluorescence imaging with excitation wavelength (Ex_wl_) 633 nm and emission window 650–700 nm for Cy5, 660 nm and emission window of 670–720 nm for Cy5.25, and 670 nm with 680–730 nm as an emission window for Cy5.5. For the control staining, excitation of 405 nm and emission window of 420–470 nm (Hoechst) and excitation of 488 nm and emission window of 500–550 nm were used. Confocal image acquisition and processing were conducted using LASX software (Leica Application Software Suite 4.8).

For the confocal evaluation of ICG, a Zeiss LSM980 Airscan 2 confocal microscope was used (Carl Zeiss Industrielle Messtechnik GmbH, Oberkochen, Germany). Cells were first incubated with LysoTracker and Hoechst, as mentioned above, following incubation with 1 μM ICG solution in DMEM for 10 min.

A full‐spectrum flow cytometer, Aurora 3L (Cytek Biosciences, Fremont, CA, US), was used to assess the total cellular uptake of the cyanine derivatives in the HC‐04.J7 cell line. After incubation with 100 nM (dyes **1**‐**3**) or 1 µM (dyes **4**‐**9**) of cyanine dye solution in DMEM for 10 min, cells were trypsinized. Following two washes with PBS, the cell suspension was measured by a flow cytometer. At least 50,000 events were recorded per sample. To evaluate the mean fluorescence intensity (MFI), data were analyzed using FlowJoTM software (BD Biosciences, Franklin Lakes, NJ, USA) on a gated population of single cells. The significance of the results was determined using the nonparametric one‐way ANOVA test in GraphPad Prism software (V10.2.3, Boston, Massachusetts, USA, www.graphpad.com).

To assess the presence of OATP1B1 (liver‐specific influx transporters important in initial substrate recognition and translocation across the membrane) [39], Western blot analysis was performed on HC‐04.J7 and Ge*β*3 following a previously published protocol with slight adaptations for the institutional setup [25]. AB312838 (Abcam, Cambridge, UK) antibodies were used for OATP1B1, while GAPDH (MA5−35 235, Invitrogen, Waltham, Massachusetts, USA) served as a loading control.

### 5.6 Cluster Analysis

Data processing and analysis were performed using the Python programing language (version 3.10+). The Spearman correlation coefficient (r) was computed for all pairs of the numerical variables using the *.corr*() method with the default Spearman method, where +1 (perfect monotonic relationship) to −1 (perfect negative monotonic relationship), with 0 indicating no monotonic relationship. The resulting correlation matrix was visualized using a clustered heatmap generated with the seaborn (version 0.12.2) library's *clustermap*() function.

The data processing, normalization, and visualization steps were performed using Python (version 3.10+). The numerical features selected for analysis were normalized to a standardized range of [−1, 1] using the Min–Max Scaling technique. A feature Serum binding underwent a specialized normalization process relative to a defined range (0–100). The scaling was adjusted such that a value of 0 was mapped to −1 and a value of 100 was mapped to +1, with intermediate values scaled proportionally. To visually represent the relationships between the features and the dyes, the seaborn *clustermap*() (version 0.12.2) function was used.

## Supporting Information

Additional supporting information can be found online in the Supporting Information section.

## Funding

This research was funded by the Research Foundation of Flanders (Grant no. FWOS000922N) and an NWO‐TTW‐VICI (TTW BGT16141) and NWO‐KIC (KICH1.ST03.21.030) grant supported by the Dutch Research Council.

## Conflicts of Interest

The authors declare no conflicts of interest.

## Supporting information

Supplementary Material

## Data Availability

The data that support the findings of this study are available in the supplementary material of this article.
