# Supplementary material for: Structure–Activity Relationship of Cyanine Dyes in Relation to the Hepatic Uptake and Excretion
Source: Chembiochem. 2026 Apr 17;27(8):e202500762. doi: 10.1002/cbic.202500762 (PMC13089939; doi:10.1002/cbic.202500762)
Supplement: Supplementary file 1 — Supplementary Material [file CBIC-27-e202500762-s001.pdf]

Supporting Information

Structure–activity relationship of Cyanine dyes in relation to the hepatic uptake and excretion

Nataliia Berehova[1], Emily de Bock[1], Maarten P. van Meerbeek[1], Chanel M. Naar[1,2], Thom van den Eng[1], Nikita A. Bouw[1], *Tessa Buckle[1], Fijs W.B. van Leeuwen[1]

[1]Interventional Molecular Imaging Laboratory (IMI), Leiden University Medical Center, Leiden, The Netherlands.
[2]Leiden University Center for Infectious Diseases (LUCID), Leiden University Medical Center, Leiden, The Netherlands.

*Correspondence t.buckle@lumc.nl

Table of contents

General synthetic procedures of dyes **1**-**9** 2
General synthetic procedure 1: Synthesis of symmetrical cyanine-5 dyes **1**, **3**, **7**, and **9**.......... 2
General synthetic procedure 2: Synthesis of asymmetrical cyanine-5 dyes **2**, **4**-**6**, and **8**. 2
Synthesis of dye **1**.......... 3
Synthesis of dye **2**.......... 3
Synthesis of dye **3**.......... 4
Synthesis of dye **4**.......... 4
Synthesis of dye **5**.......... 5
Synthesis of dye **6**.......... 5
Synthesis of dye **7**.......... 6
Synthesis of dye **8** 6
Synthesis of dye **9**.......... 7
^{1}H-NMR and ^{13}C-NMR spectra of the synthesized dyes **1**-**9** 8
Mass analysis of cyanine dyes **1** - **9**.......... 17
Figure S28. Assessment of the presence of liver-specific influx transporters OATP1B1 in hepatocytes HC-04.J7 and control epithelial cells Geβ3.......... 20
Table S1. Chemical and optical properties of ICG. 20
Figure S29. Confocal evaluation of the ICG in the HC-04.J7 cell line. 20
References.......... 21

General synthetic procedures of dyes 1-9

General synthetic procedure 1: Synthesis of symmetrical cyanine-5 dyes **1**, **3**, **7**, and **9**.
The corresponding indole building block (1 eq) was dissolved in absolute ethanol following the addition of malonaldehyde dianilide HCl (0.5 eq) and sodium acetate (1.2 eq). The reaction mixture was heated to 80°C and left to stir for 7 hours. After this time, the heating was removed, and the reaction mixture was stirred for 48 hours at room temperature. Upon completion, the solvent was removed, and the reaction mixture was purified by flash chromatography, followed by purification by preparative HPLC.

General synthetic procedure 2: Synthesis of asymmetrical cyanine-5 dyes **2**, **4-6**, and **8.**

2A: Corresponding first indole building block (1 eq) was dissolved in 1:1 acetic acid: acetic anhydride following the addition of malonaldehyde dianilide HCl (1.2 eq.) and was heated to 90°C overnight. After an overnight reaction, heating was removed, and the reaction mixture was cooled to room temperature. The solution was added dropwise to a 20-fold reaction volume of vigorously stirring diethyl ether. The resulting precipitate was collected through centrifugation, and the formed pellet was washed with diethyl ether twice more. The crude intermediate product was dried *in vacuo* and used as is in the following step.

2B: The corresponding second indole building block (1 eq) was added to the reaction vessel following the addition of previously obtained intermediate dissolved in 10 mL 3:1 pyridine: acetic anhydride. The reaction mixture was left to stir at 50°C overnight. After an overnight reaction, heating was removed, and the reaction mixture was cooled down to room temperature. The solution was added dropwise to a 20-fold reaction volume of vigorously stirring diethyl ether. The resulting precipitate was centrifuged, and the formed pellet was washed with diethyl ether twice more. The crude intermediate product was dried *in vacuo* and purified using preparative HPLC.

Synthesis of dye 1: 1,3,3-trimethyl-2-((1E,3E)-5-((E)-1,3,3-trimethylindolin-2-ylidene)penta-1,3-dien-1-yl)-3H-indol-1-ium

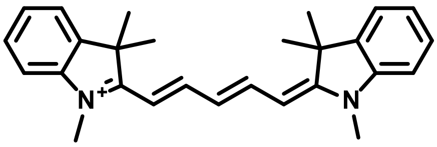

General procedure 1 was followed, where Indole-Me (600 mg; 332 µmol), malonaldehyde dianilide HCl (257.7 mg; 166 µmol), and sodium acetate (32 mg; 398.4 µmol) were added to 7 mL of absolute ethanol. The crude product was purified through flash chromatography, 100:0 DCM: MeOH → 98:2 DCM: MeOH, followed by purification by preparative HPLC, employing a gradient of 45 to 95% acetonitrile over 31 min. Lyophilization of product-containing fractions yielded the title compound as a dark blue solid (237.1 mg, 30.6% isolated yield). Mass calculated ($C_{27}H_{31}N_2^+$) 383.6; found MS (ES^+): m/z 383.6. ^{1}H-NMR (400 MHz, DMSO) δ 8.31 (dd, J = 13.9, 12.4 Hz, 2H), 7.60 (dd, J = 7.4, 1.0 Hz, 2H), 7.40 – 7.34 (m, 4H), 7.22 (dt, J = 7.5, 4.2 Hz, 2H), 6.53 (t, J = 12.4 Hz, 1H), 6.26 (d, J = 13.9 Hz, 2H), 3.61 (s, 6H), 1.66 (s, 12H). ^{13}C NMR (101 MHz, DMSO) δ 173.54, 154.43, 143.25, 141.46, 128.76, 125.55, 125.09, 122.73, 111.42, 103.68, 49.25, 31.75, 31.49, 27.43.

Synthesis of dye 2: 1,3,3-trimethyl-2-((1E,3E,5E)-5-(1,1,3-trimethyl-1,3-dihydro-2H-benzo[e]indol-2-ylidene)penta-1,3-dien-1-yl)-3H-indol-1-ium

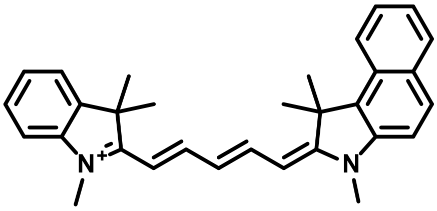

General procedure 2 was followed.

2A: indole-Me (100 mg; 573.7 µmol) and malonaldehyde dianilide HCl (178.2 mg; 688.5 µmol) were dissolved in 3 mL 1:1 acetic acid: acetic anhydride.

2B: The freshly obtained intermediate was mixed with (Ar)indole-Me (128.7 mg, 573.7 µmol) in 10 mL 3:1 pyridine: acetic anhydride. The resulting crude mixture was purified by preparative HPLC, employing a gradient of 45% to 95% acetonitrile over 31 minutes. Lyophilization of product-containing fractions yielded the title compound as a dark blue solid (85.4 mg, 34.3% isolated yield). Mass calculated ($C_{31}H_{33}N_2^+$) 433.6; found MS (ES^+): m/z 433.6. ^{1}H-NMR (400 MHz, DMSO) δ 8.45 (ddd, J = 14.0, 12.3 Hz, 1H), 8.34 (t, J = 13.1 Hz, 1H), 8.25 (dd, J = 8.6, 1.1 Hz, 1H), 8.12 – 8.03 (m, 2H), 7.77 (d, J = 8.8 Hz, 1H), 7.68 (ddd, J = 8.4, 6.8, 1.4 Hz, 1H), 7.61 (dd, J = 7.2, 1.0 Hz, 1H), 7.52 (ddd, J = 8.1, 6.9, 1.0 Hz, 1H), 7.44 – 7.34 (m, 2H), 7.24 (td, J = 7.1, 1.5 Hz, 1H), 6.57 (t, J = 12.3 Hz, 1H), 6.35 (d, J = 14.0 Hz, 1H), 6.26 (d, J = 13.8 Hz, 1H), 3.75 (s, 3H), 3.60 (s, 3H), 1.94 (s, 6H), 1.70 (s, 6H). ^{13}C NMR (101 MHz, DMSO) δ 175.15, 173.05, 173.02, 153.89, 153.83, 143.34, 141.37, 140.85, 133.60, 131.83, 130.68, 130.41, 128.80, 128.22, 127.95, 125.66, 125.28, 124.94, 122.75, 122.63, 112.13, 111.29, 103.78, 103.38, 51.22, 49.15, 40.64, 40.59, 40.43, 40.38, 40.17, 39.96, 39.75, 39.54, 39.34, 32.03, 31.43, 27.54, 27.04.

Synthesis of dye 3: 1,1,3-trimethyl-2-((1E,3E,5E)-5-(1,1,3-trimethyl-1,3-dihydro-2H-benzo[e]indol-2-ylidene)penta-1,3-dien-1-yl)-1H-benzo[e]indol-3-ium

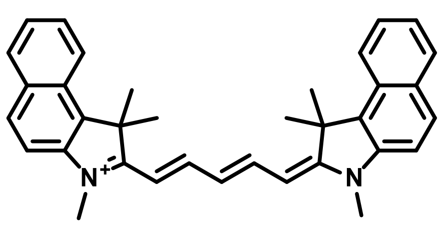

General procedure 1 was followed, where (Ar)indole-Me (100 mg; 445.8 µmol), malonaldehyde dianilide HCl (57.7 mg; 222.9 µmol), and sodium acetate (43.9 mg; 535 µmol) were added to 5 mL of absolute ethanol. The crude product was purified through flash chromatography. 95:5:1 DCM:MeOH:AcOH. The resulting crude mixture was subsequently purified by preparative HPLC, employing a gradient of 45% to 95% acetonitrile over 31 minutes. Lyophilization of product-containing fractions yielded the title compound as a dark blue solid (29.6 mg, 13.7% isolated yield). Mass calculated ($C_{35}H_{35}N_2^+$) 483.7; found MS (ES$^+$): m/z 483.5. ^{1}H-NMR (400 MHz, DMSO) δ 8.46 (ddd, *J* = 13.9, 12.3 Hz, 2H), 8.26 (d, *J* = 8.5 Hz, 2H), 8.12 – 8.04 (m, 4H), 7.76 (d, *J* = 8.9 Hz, 2H), 7.70 – 7.64 (m, 2H), 7.52 (ddd, *J* = 8.1, 6.8, 1.0 Hz, 2H), 6.60 (t, *J* = 12.3 Hz, 1H), 6.34 (d, *J* = 13.9 Hz, 2H), 3.74 (s, 6H), 1.96 (s, 12H). ^{13}C NMR (101 MHz, DMSO) δ 174.61, 153.32, 140.92, 133.41, 131.75, 130.66, 130.41, 128.19, 128.00, 125.76, 125.19, 122.62, 112.06, 103.49, 51.10, 31.94, 27.11.

Synthesis of dye 4: 4-((E)-3,3-dimethyl-2-((2E,4E)-5-(1,3,3-trimethyl-3H-indol-1-ium-2-yl)penta-2,4-dien-1-ylidene)indolin-1-yl)butyl sulfite

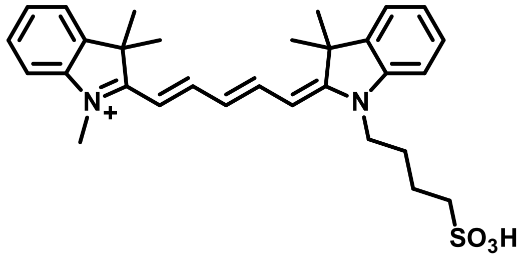

General procedure 2 was followed.

2A: The indole-Me (100 mg; 573.7 µmol) and malonaldehyde dianilide HCl (178.2 mg; 688.5 µmol) were dissolved in 3 mL 1:1 acetic acid: acetic anhydride.

2B: The intermediate was mixed with indole-SO_3 (169.5 mg, 573.7 µmol) in 10 mL 3:1 pyridine: acetic anhydride. After overnight refluxing, the mixture was precipitated and concentrated *in vacuo*. The resulting crude was purified by preparative HPLC, employing a gradient of 35 to 95% acetonitrile over 36 min. Lyophilization of product-containing fractions yielded the title compound as a dark blue solid (81.3 mg, 28.1% isolated yield). Mass calculated ($C_{30}H_{37}N_2O_3S^+$) 505.7; found MS (ES$^+$): m/z 505.6. 1H NMR (400 MHz, DMSO) δ 8.38 – 8.27 (m, 2H), 7.64 – 7.58 (m, 2H), 7.46 – 7.35 (m, 4H), 7.27 – 7.20 (m, 2H), 6.57 (t, J = 12.4 Hz, 1H), 6.41 (d, J = 13.8 Hz, 1H), 6.26 (d, J = 13.8 Hz, 1H), 4.11 (t, J = 7.2 Hz, 2H), 3.60 (s, 3H), 2.49 (d, J = 6.3 Hz, 2H), 1.87 – 1.70 (m, 5H), 1.67 (s, 12H). ^{13}C NMR (101 MHz, DMSO) δ 173.37, 173.12, 154.66, 154.63, 154.24, 143.29, 142.47, 141.60, 141.43, 128.91, 128.79, 125.89, 125.14, 125.02, 122.85, 122.74, 111.71, 111.39, 103.92, 103.58, 51.09, 49.34, 49.21, 43.84, 31.78, 31.48, 27.61, 27.51, 26.44, 22.94.

Synthesis of dye 5: 4-((E)-1,1-dimethyl-2-((2E,4E)-5-(1,3,3-trimethyl-3H-indol-1-ium-2-yl)penta-2,4-dien-1-ylidene)-1,2-dihydro-3H-benzo[e]indol-3-yl)butyl sulfite

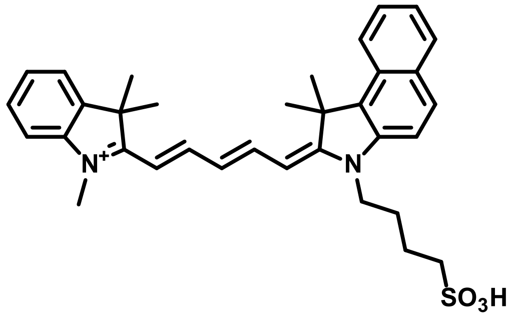

General procedure 2 was followed.

2A: The indole-Me (100 mg; 573.7 µmol) and malonaldehyde dianilide HCl (178.2 mg; 688.5 µmol) were dissolved in 3 mL 1:1 acetic acid: acetic anhydride.

2B: The intermediate was mixed with (Ar)indole-SO_3 (198.2 mg, 573.7 µmol) in 10 mL 3:1 pyridine: acetic anhydride. The resulting crude was purified by preparative HPLC, employing a gradient of 35 to 95% acetonitrile over 36 min. Lyophilization of product-containing fractions yielded the title compound as a dark blue solid (144.6 mg, 45.4% isolated yield). Mass calculated ($C_{34}H_{39}N_2O_3S^+$) 555.8; found MS (ES^+): m/z 555.6. ^{1}H-NMR (400 MHz, DMSO) δ 8.43 (dd, J = 13.9, 12.2 Hz, 1H), 8.32 (t, J = 13.1 Hz, 1H), 8.23 (d, J = 8.6 Hz, 1H), 8.09 – 8.01 (m, 2H), 7.79 (d, J = 8.9 Hz, 1H), 7.66 (ddd, J = 8.4, 6.8, 1.3 Hz, 1H), 7.58 (d, J = 7.4 Hz, 1H), 7.49 (ddd, J = 8.0, 6.9, 1.0 Hz, 1H), 7.41 – 7.33 (m, 2H), 7.24 – 7.18 (m, 1H), 6.59 (t, J = 12.3 Hz, 1H), 6.48 (d, J = 14.0 Hz, 1H), 6.23 (d, J = 13.7 Hz, 1H), 4.26 (t, J = 7.4 Hz, 2H), 3.59 (s, 3H), 2.55 (t, J = 7.1 Hz, 2H), 1.92 (s, 6H), 1.89 – 1.74 (m, 5H), 1.67 (s, 6H). ^{13}C NMR (101 MHz, DMSO) δ 174.67, 172.84, 154.01, 153.72, 143.34, 141.34, 140.10, 133.78, 131.82, 130.79, 130.38, 128.76, 128.16, 127.99, 125.98, 125.27, 124.83, 122.71, 122.58, 112.27, 111.22, 103.92, 103.33, 51.26, 51.08, 49.09, 44.07, 31.77, 31.39, 27.55, 27.17, 26.72, 22.88.

Synthesis of dye 6: 4-((E)-1,1-dimethyl-2-((2E,4E)-5-(1,1,3-trimethyl-1H-benzo[e]indol-3-ium-2-yl)penta-2,4-dien-1-ylidene)-1,2-dihydro-3H-benzo[e]indol-3-yl)butyl sulfite

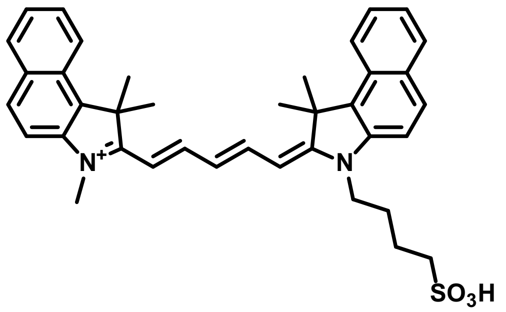

General procedure 2 was followed.

2A: The (Ar)indole-Me (100 mg; 445.8 µmol) and malonaldehyde dianilide HCl (138.5 mg; 535.0 µmol) were dissolved in 3 mL 1:1 acetic acid: acetic anhydride.

2B: The intermediate was mixed with (Ar)indole-SO_3 (154.0 mg, 445.8 µmol) in 10 mL 3:1 pyridine: acetic anhydride. The resulting crude was purified by preparative HPLC, employing a gradient of 45 to 95% acetonitrile over 31 min. Lyophilization of product-containing fractions yielded the title compound as a dark blue solid (189.3 mg, 70.2% isolated yield). Mass calculated ($C_{38}H_{41}N_2O_3S^+$) 605.8; found MS (ES^+): m/z 605.2. ^{1}H-NMR (400 MHz, DMSO) δ 8.44 (t, J = 13.1 Hz, 2H), 8.44 (t, J = 13.1 Hz, 1H), 8.44 (t, J = 13.1 Hz, 1H), 8.24 (d, J = 8.5 Hz, 2H), 8.05 (ddd, J = 10.3, 8.5, 1.8 Hz, 4H), 7.76 (dd, J = 16.1, 8.9 Hz, 2H), 7.67 (ddt, J = 8.3, 6.7, 1.4 Hz, 2H), 7.54 – 7.45 (m, 2H), 6.62 (t, J = 12.3 Hz, 1H), 6.46 (d, J = 13.9 Hz, 1H), 6.31 (d, J = 13.9 Hz, 1H), 4.25 (t, J = 7.4 Hz, 2H), 3.73 (s, 3H), 2.55 (t, J = 7.0 Hz, 2H), 1.94 (s, 12H), 1.89 – 1.75 (m, 4H). ^{13}C NMR (101 MHz, DMSO) δ 174.20, 173.89, 153.24,

152.90, 140.70, 139.95, 133.36, 133.11, 131.51, 131.47, 130.54, 130.37, 130.15, 127.90, 127.80, 127.78, 125.82, 124.95, 124.87, 122.36, 122.34, 111.97, 111.80, 103.40, 103.19, 67.17, 50.91, 50.86, 50.80, 43.77, 31.66, 31.54, 27.00, 26.89, 26.45, 22.67.

Synthesis of dye 7: 4-((E)-2-((2E,4E)-5-(3,3-dimethyl-1-(4-(sulfinatooxy)butyl)-3H-indol-1-ium-2-yl)penta-2,4-dien-1-ylidene)-3,3-dimethylindolin-1-yl)butyl sulfite

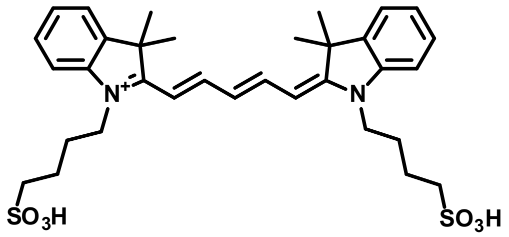

General procedure 1 was followed, where indole-SO_3 (100 mg; 338.5 µmol), malonaldehyde dianilide HCl (43.8 mg; 169.3 µmol), and sodium acetate (33.3 mg; 306.2 µmol) were added to 5 mL of absolute ethanol. The crude product was purified through flash chromatography. 100:0 DCM: MeOH → 70:30 DCM: MeOH. The resulting crude mixture was subsequently purified by preparative HPLC, employing a gradient of 5% to 95% acetonitrile over 51 minutes. Lyophilization of product-containing fractions yielded the title compound as a dark blue solid (25.6 mg, 12.0% isolated yield). Mass calculated ($C_{33}H_{43}N_2O_6S_2^+$) 627.8; found MS (ES^+): m/z 627.4. ^{1}H-NMR (400 MHz, DMSO) δ 8.33 (t, J = 13.1 Hz, 2H), 7.61 (dd, J = 7.4, 1.1 Hz, 2H), 7.45 – 7.36 (m, 4H), 7.24 (td, J = 7.2, 1.3 Hz, 2H), 6.60 (t, J = 12.4 Hz, 1H), 6.37 (d, J = 13.8 Hz, 2H), 4.11 (t, J = 7.2 Hz, 4H), 2.55 (t, J = 7.2 Hz, 4H), 1.85 – 1.70 (m, 10H), 1.68 (s, 12H). ^{13}C NMR (101 MHz, DMSO) δ 172.95, 154.49, 142.53, 141.57, 128.90, 126.16, 125.07, 122.84, 111.66, 103.78, 51.17, 49.31, 43.76, 31.78, 27.65, 26.44, 22.81.

Synthesis of dye 8: 4-((E)-1,1-dimethyl-2-((2E,4E)-5-(1,3,3-trimethyl-3H-indol-1-ium-2-yl)penta-2,4-dien-1-ylidene)-1,2-dihydro-3H-benzo[e]indol-3-yl)butyl sulfite

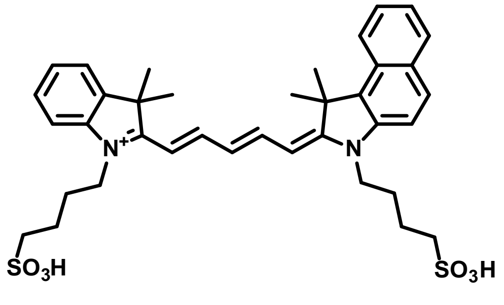

General procedure 2 was followed.

2A: The indole-SO_3 (100 mg; 338.52 µmol) and malonaldehyde dianilide HCl (105.1 mg; 406.2 µmol) were dissolved in 3 mL 1:1 acetic acid: acetic anhydride.

2B: The intermediate was mixed with (Ar)indole-SO_3 (116.9 mg, 338.5 µmol) in 10 mL 3:1 pyridine: acetic anhydride. The resulting crude was purified by preparative HPLC, employing a gradient of 5 → 95% acetonitrile over 51 min. Lyophilization of product-containing fractions yielded the title compound as a dark blue solid (112.1 mg, 48.9% isolated yield). Mass calculated ($C_{37}H_{45}N_2O_6S_2^+$) 677.9; found MS (ES^+): m/z 677.4. 1H NMR (600 MHz, DMSO) δ 8.44 (t, J = 13.1 Hz, 1H), 8.34 (t, J = 13.1 Hz, 1H), 8.24 (d, J = 8.5 Hz, 1H), 8.10 – 8.04 (m, 2H), 7.79 (d, J = 8.9 Hz, 1H), 7.70 – 7.65 (m, J = 8.3, 6.8, 1.3 Hz, 2H), 7.60 (dd, 1H), 7.54 – 7.49 (m, J = 8.0, 6.8, 1.0 Hz, 1H), 7.44 – 7.37 (m, 3H), 7.23 (td, J = 7.2, 1.4 Hz, 1H), 6.64 (t, J = 12.3 Hz, 1H), 6.45 (d, J = 13.9 Hz, 1H), 6.36 (d, J = 13.7 Hz, 1H), 4.25 (t, J = 7.6 Hz, 2H), 4.10 (t, J = 7.5 Hz, 2H), 2.53 (t, 2H), 1.95 (s, 6H), 1.86 – 1.72 (m, 10H), 1.70 (s, 6H). ^{13}C NMR (151 MHz, DMSO) δ 174.06, 171.93, 153.46, 153.36, 142.16, 141.00, 139.71, 133.25, 131.33, 130.30, 129.92, 128.40,

127.67, 127.54, 125.78, 124.77, 124.40, 122.32, 122.12, 111.80, 111.03, 103.32, 103.05, 50.75, 50.69, 48.72, 43.58, 43.26, 31.30, 27.25, 26.76, 26.28, 25.98, 22.43, 22.39.

Synthesis of dye 9: 4-((E)-1,1-dimethyl-2-((2E,4E)-5-(1,1,3-trimethyl-1H-benzo[e]indol-3-ium-2-yl)penta-2,4-dien-1-ylidene)-1,2-dihydro-3H-benzo[e]indol-3-yl)butyl sulfite

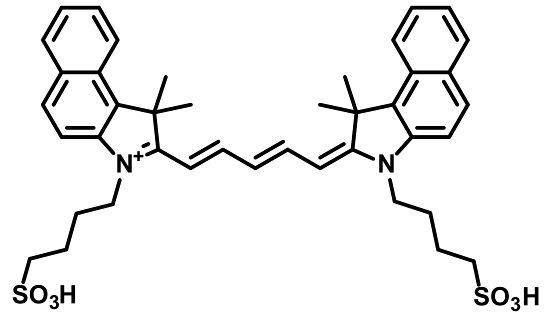

General procedure 1 was followed, where indole-SO_3 (100 mg; 289.5 µmol), malonaldehyde dianilide HCl (37.5 mg; 144.7 µmol), and sodium acetate (28.5 mg; 347.4 µmol) were added to 5 mL of absolute ethanol. The crude product was purified through flash chromatography. 80:20 DCM: MeOH → 70:30 DCM: MeOH. The resulting crude was subsequently purified by preparative HPLC, employing a gradient of 15 → 95% acetonitrile over 46 min. Lyophilization of product-containing fractions yielded the title compound as a dark blue solid (5.1 mg, 2.4% isolated yield).

General procedure 2 was followed.
2A: The (Ar)indole-SO_3 (250 mg; 723.6 µmol) and malonaldehyde dianilide HCl (93.6 mg; 361.8 µmol) were dissolved in 5 mL 1:1 acetic acid: acetic anhydride.
2B: The intermediate was mixed with (Ar)indole-SO_3 (250 mg, 723.6 µmol) in 16 mL 3:1 pyridine: acetic anhydride. The resulting crude was purified by preparative HPLC, employing a gradient of 15 → 95% acetonitrile over 46 min. Lyophilization of product-containing fractions yielded the title compound as a dark blue solid (110 mg, 20.9% isolated yield). Mass calculated ($C_{41}H_{47}N_2O_6S_2^+$) 727.9; found MS (ES^+): m/z 727.5. ^{1}H NMR (400 MHz, DMSO) δ 8.45 (t, *J* = 13.1 Hz, 2H), 8.26 (d, *J* = 8.5 Hz, 2H), 8.07 (dd, *J* = 8.5, 4.1 Hz, 4H), 7.78 (d, *J* = 9.0 Hz, 2H), 7.67 (dd, *J* = 11.3, 4.1 Hz, 2H), 7.51 (t, *J* = 7.7 Hz, 2H), 6.66 (t, *J* = 12.3 Hz, 2H), 6.44 (d, *J* = 13.8 Hz, 2H), 4.24 (t, *J* = 6.8 Hz, 4H), 2.55 (d, *J* = 7.3 Hz, 3H), 1.97 (s, 12H), 1.89 – 1.71 (m, 9H). ^{13}C NMR (151 MHz, DMSO) δ 173.55, 158.31, 158.06, 152.85, 139.78, 133.08, 131.26, 130.27, 129.92, 127.64, 127.59, 124.68, 122.10, 111.73, 103.07, 50.71, 43.49, 31.29, 26.81, 26.24, 22.36.

^{1}H-NMR and ^{13}C-NMR spectra of the synthesized dyes 1-9

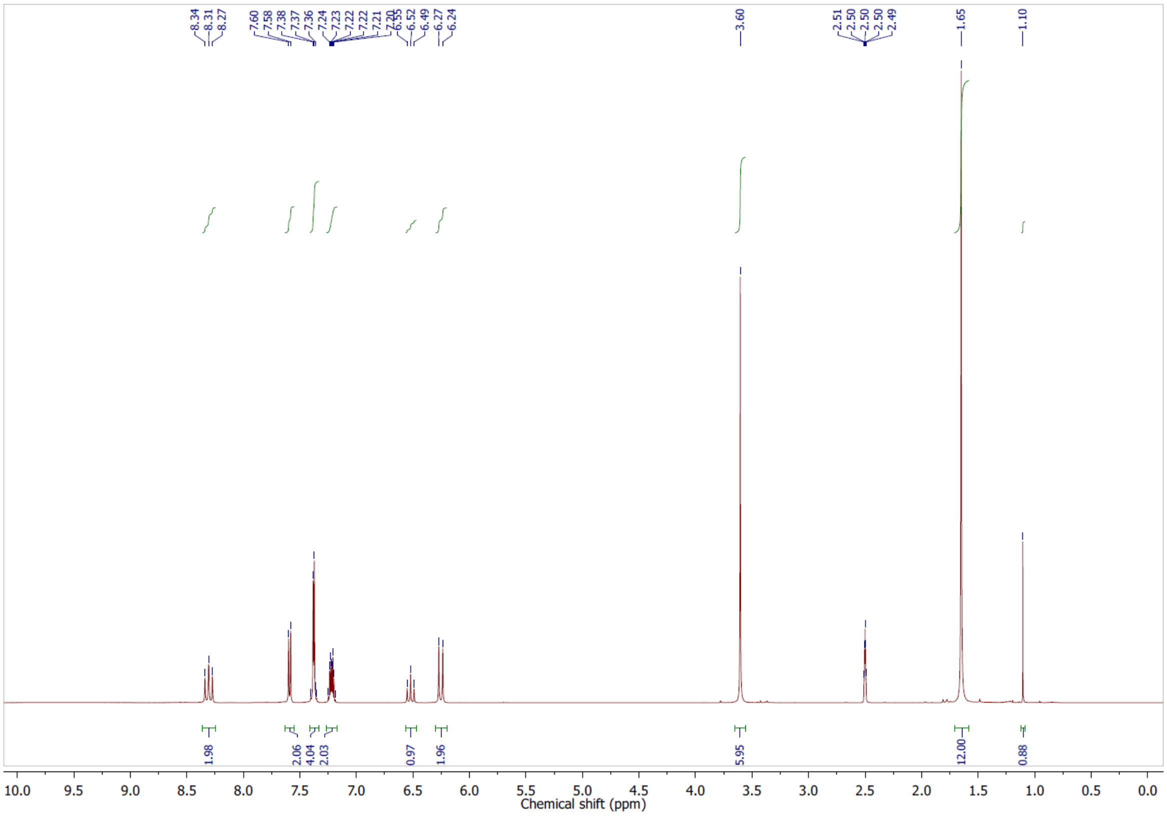

Figure S1. ^{1}H-NMR spectrum of dye **1**.

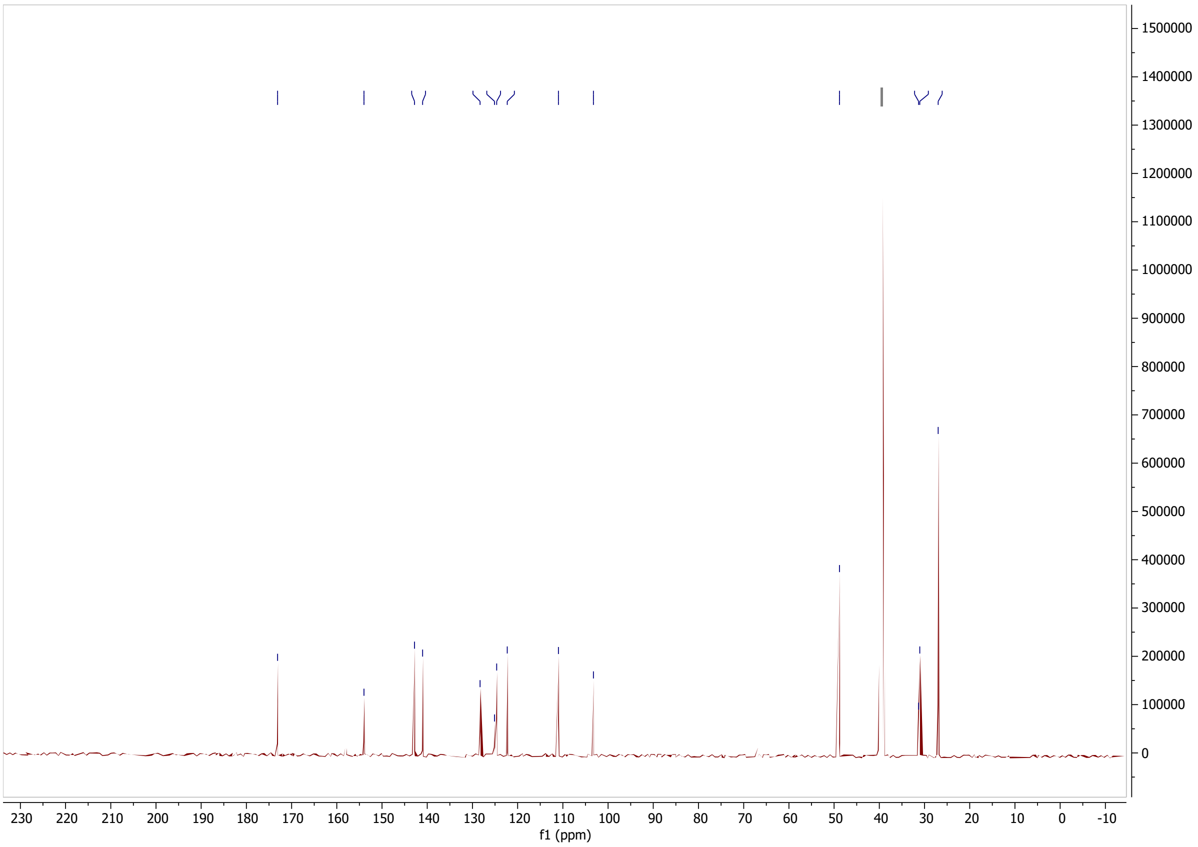

Figure S2. ^{13}C-NMR of dye **1**.

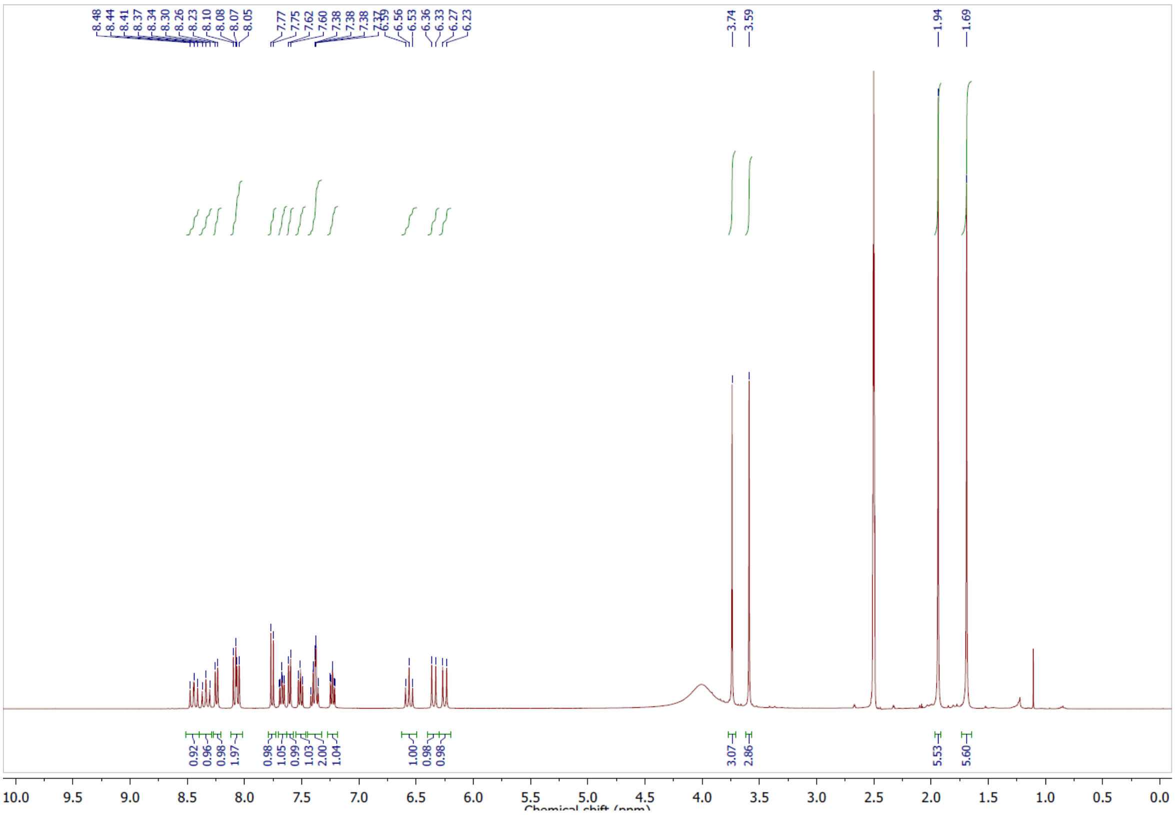

Figure S3.^{1}H-NMR spectrum of dye **2**.

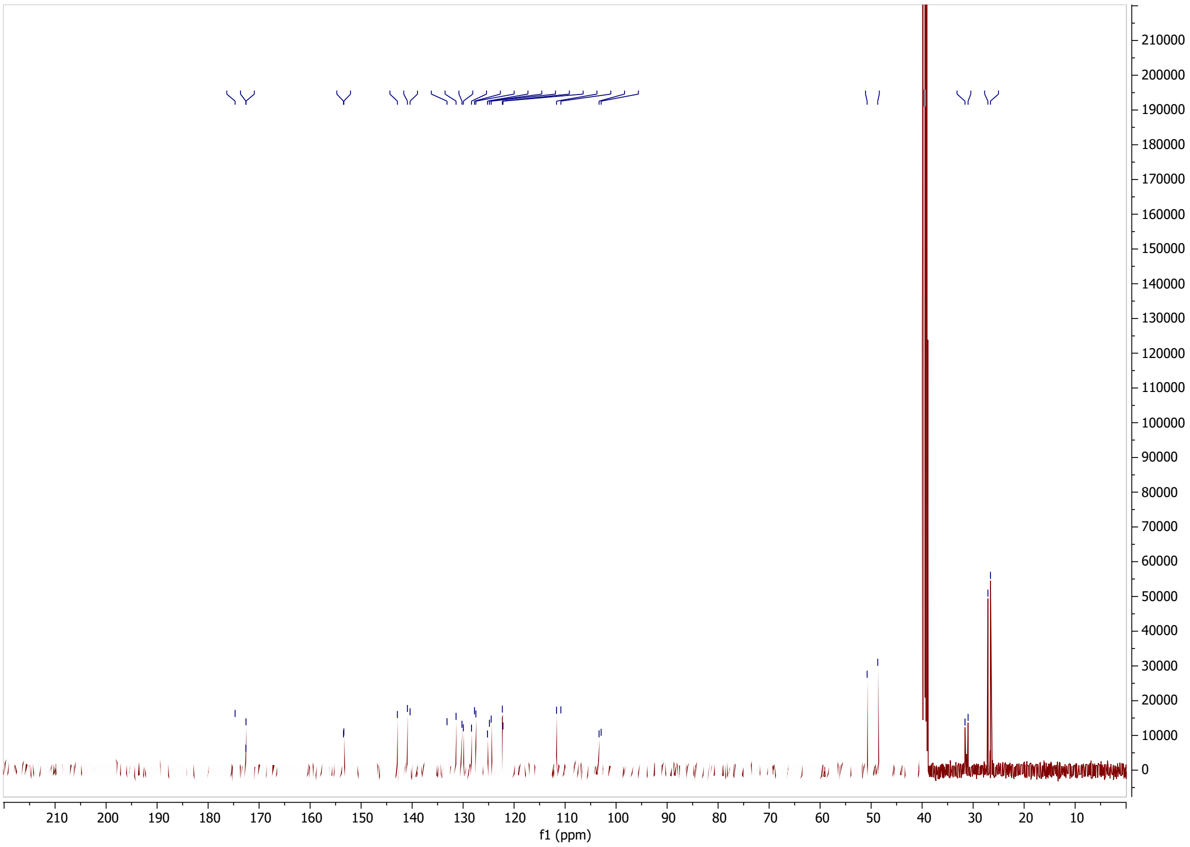

Figure S4. ^{13}C-NMR spectrum of dye **2**.

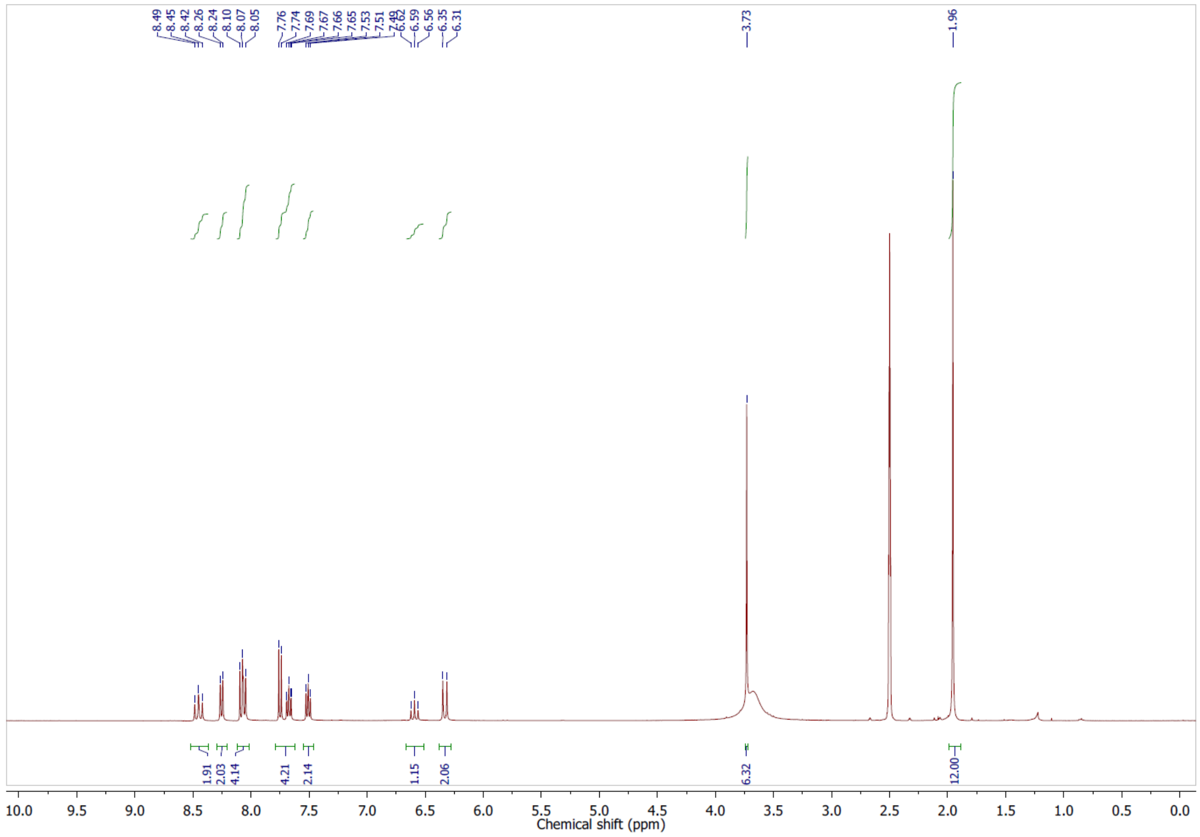

Figure S5. ^{1}H-NMR spectrum of dye **3**.

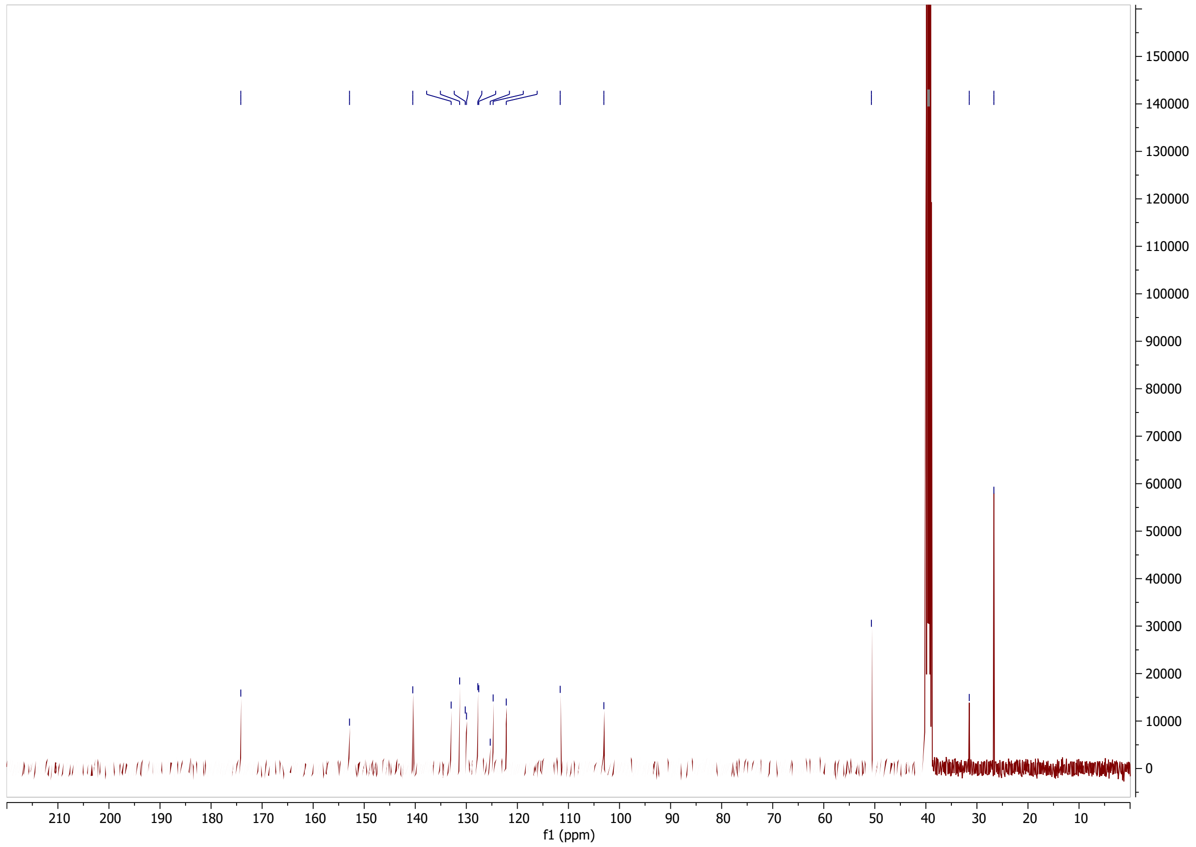

Figure S6. ^{13}C-NMR spectrum of dye **3**.

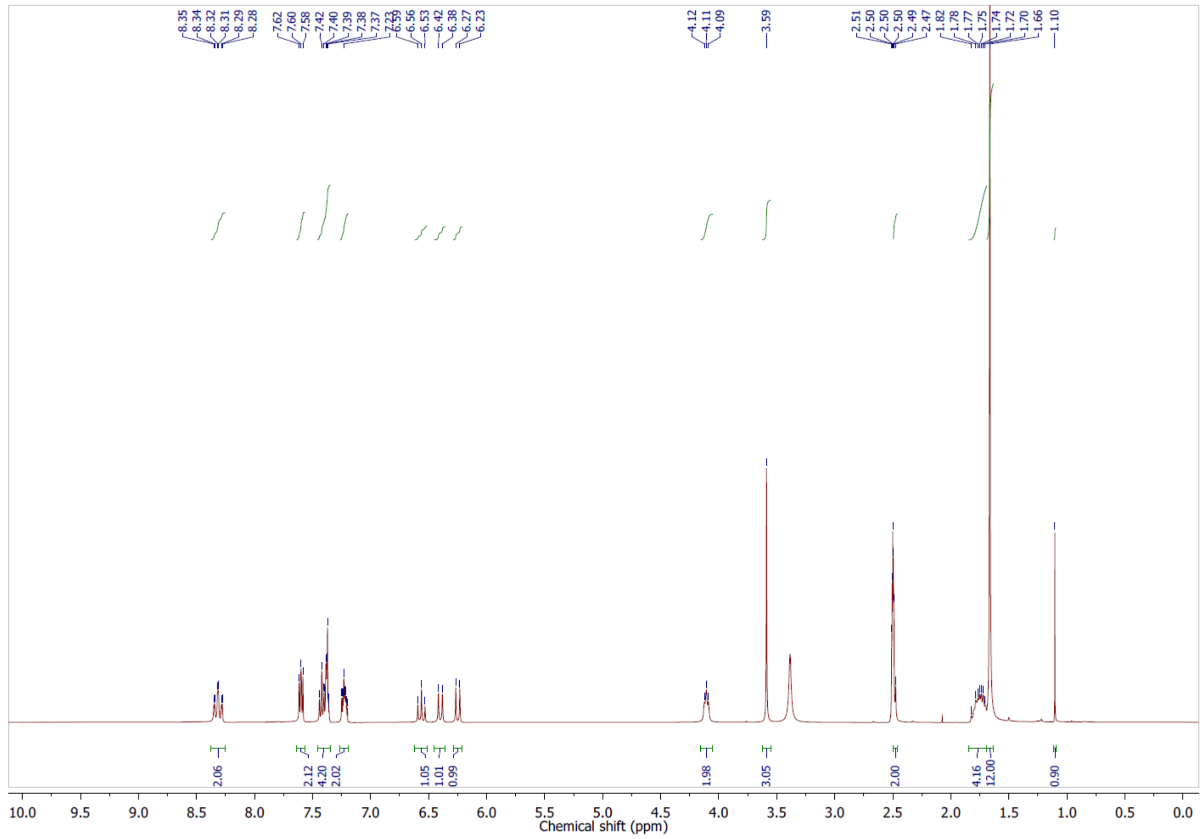

Figure S7. ^{1}H-NMR spectrum of dye **4**.

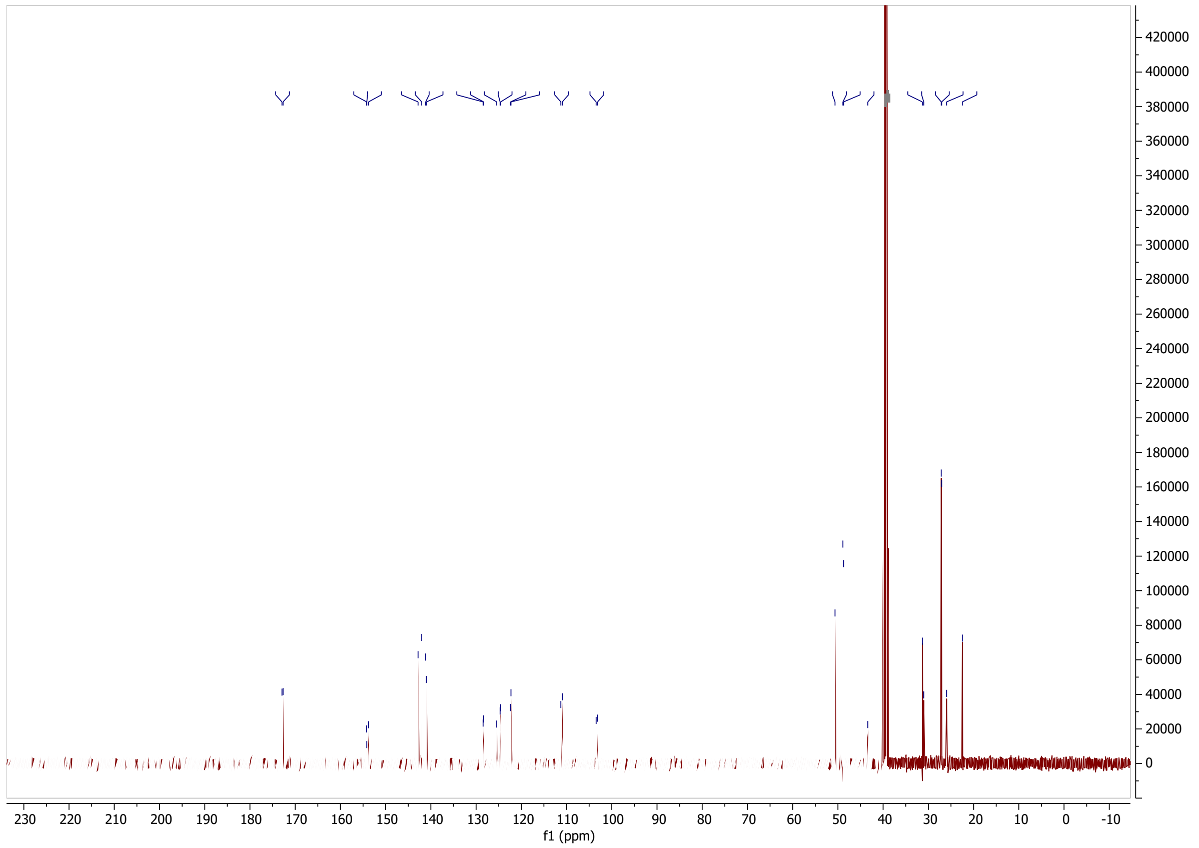

Figure S8. ^{13}C-NMR spectrum of dye **4**.

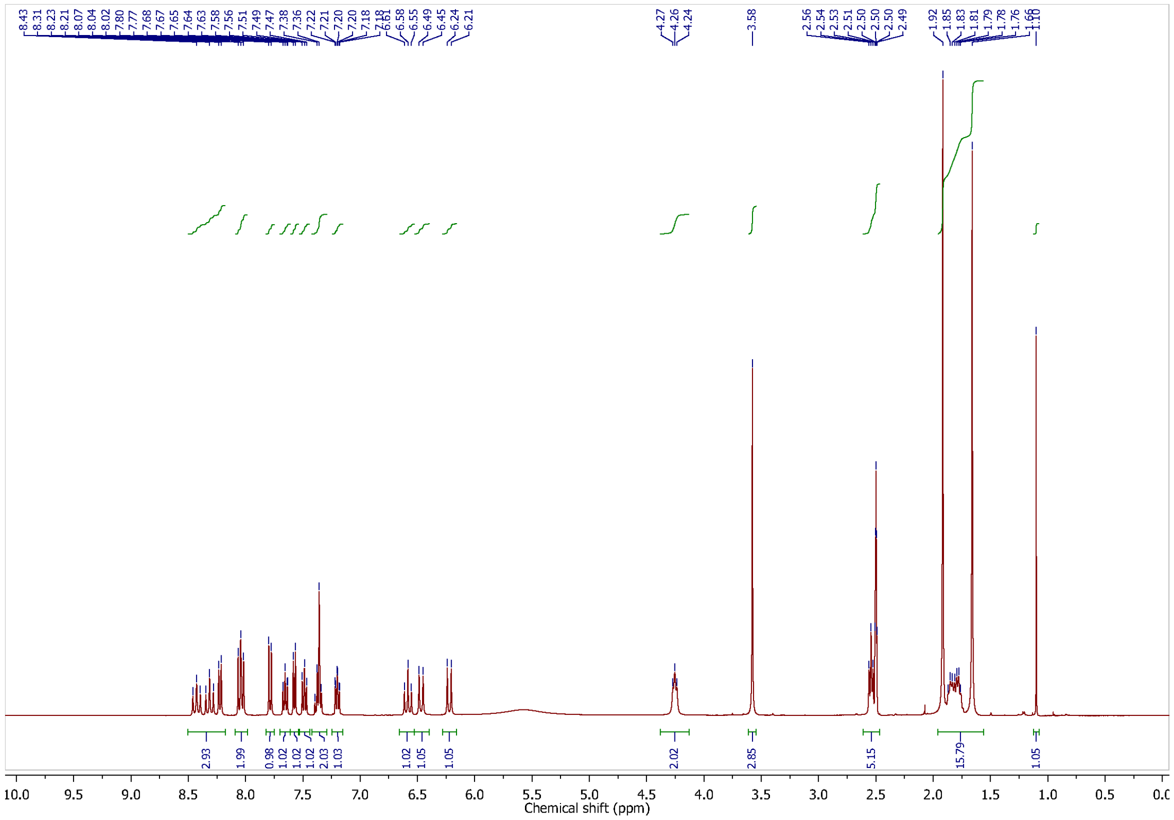

Figure S9. ^{1}H-NMR spectrum of dye **5**.

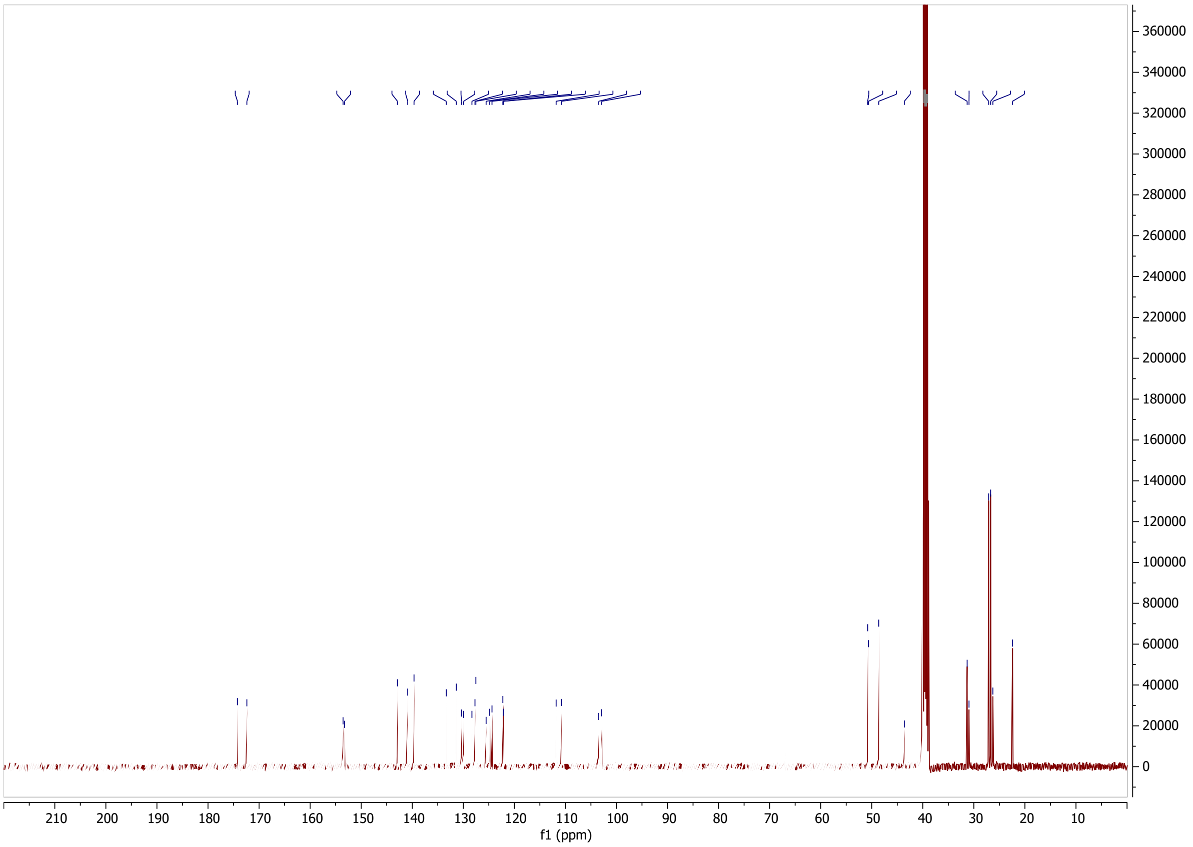

Figure S10. ^{13}C-NMR spectrum of dye **5**.

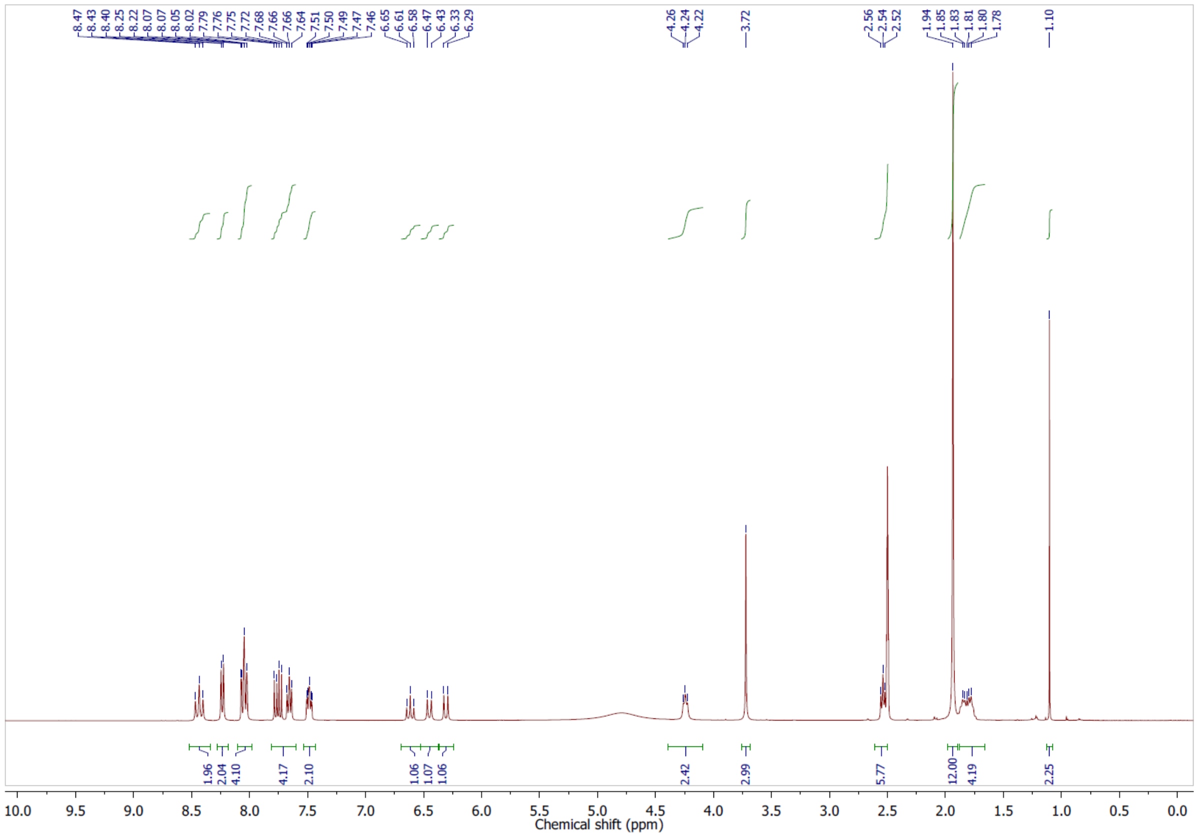

Figure S11. ^{1}H-NMR spectrum of dye **6**.

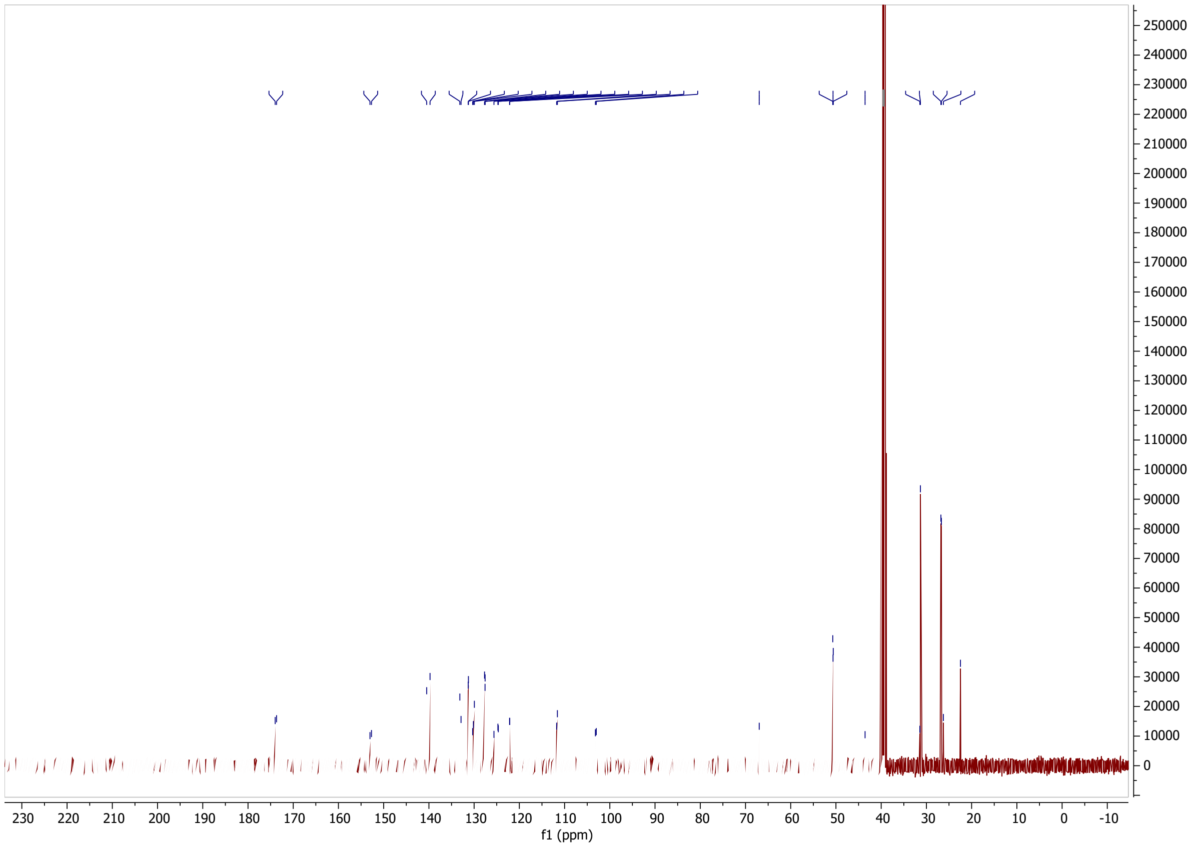

Figure S12. ^{13}C-NMR spectrum of dye **6**.

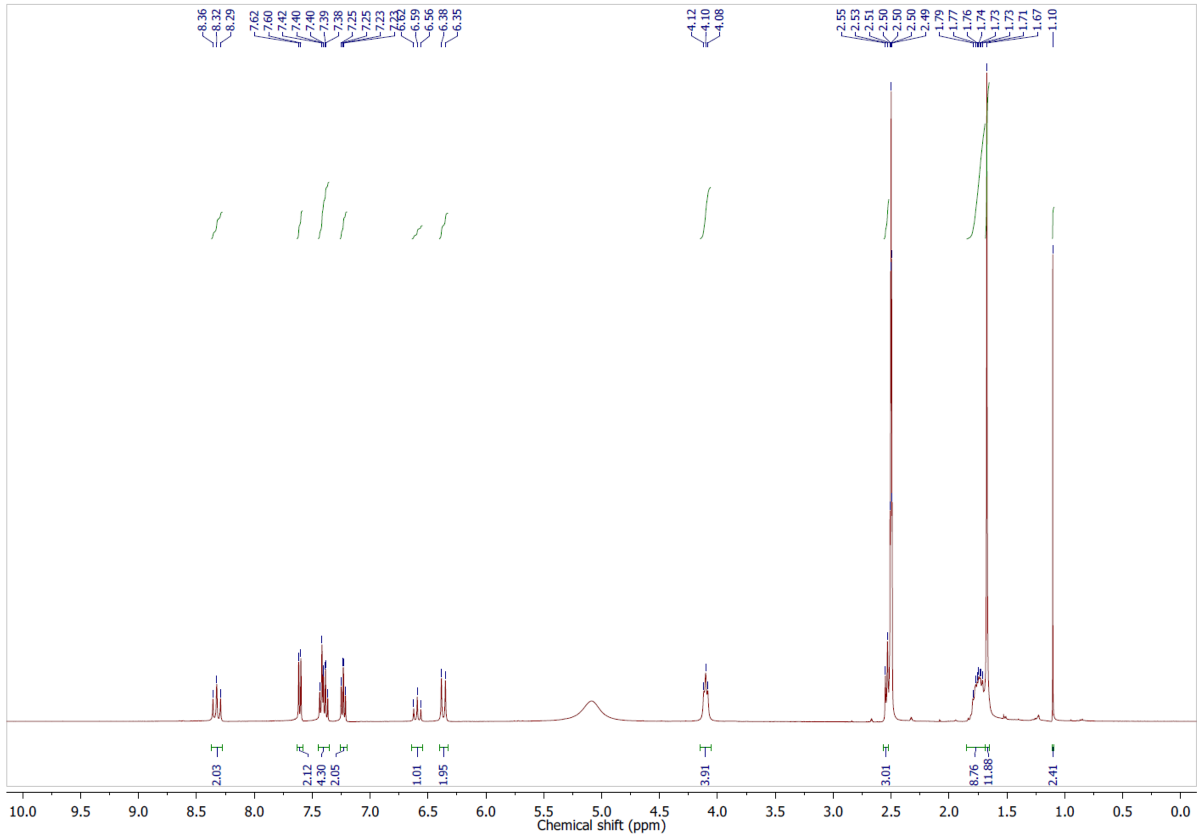

Figure S13. ^{1}H-NMR spectrum of dye **7**.

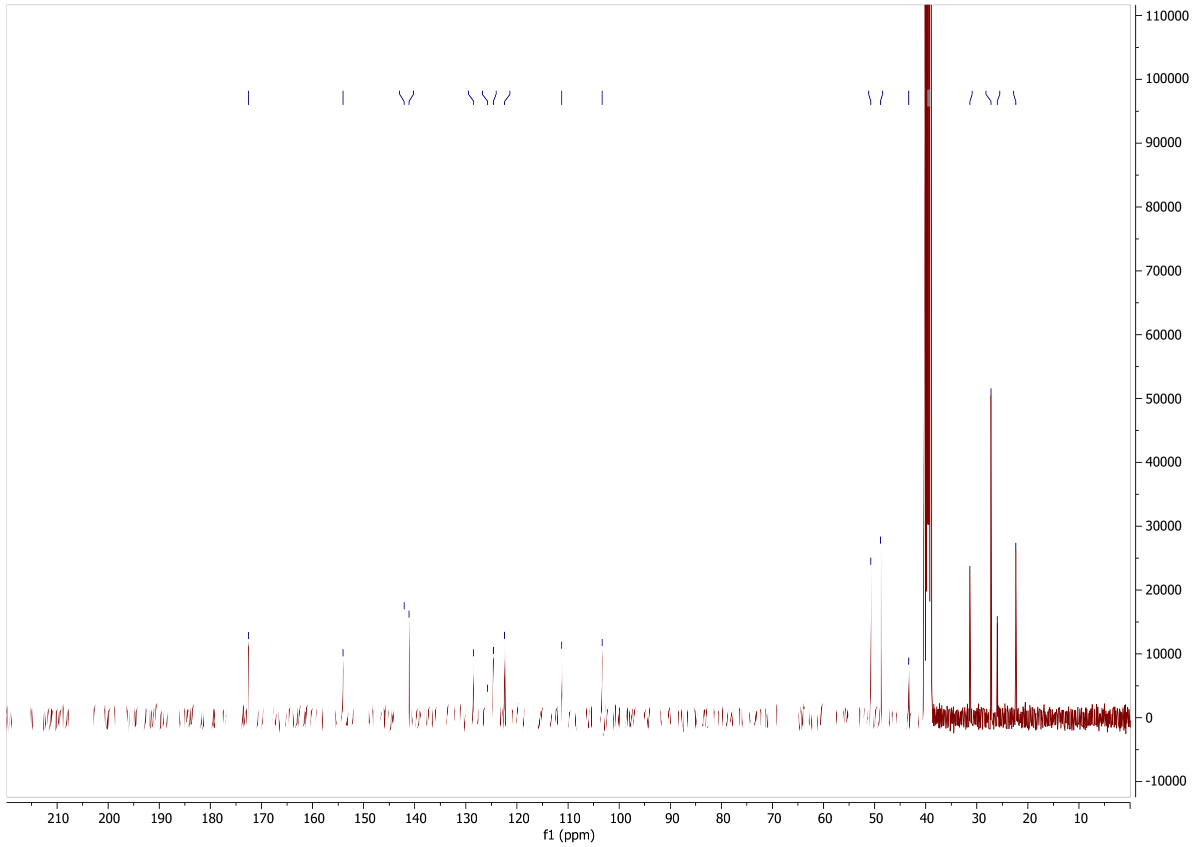

Figure S14. ^{13}C-NMR spectrum of dye **7**.

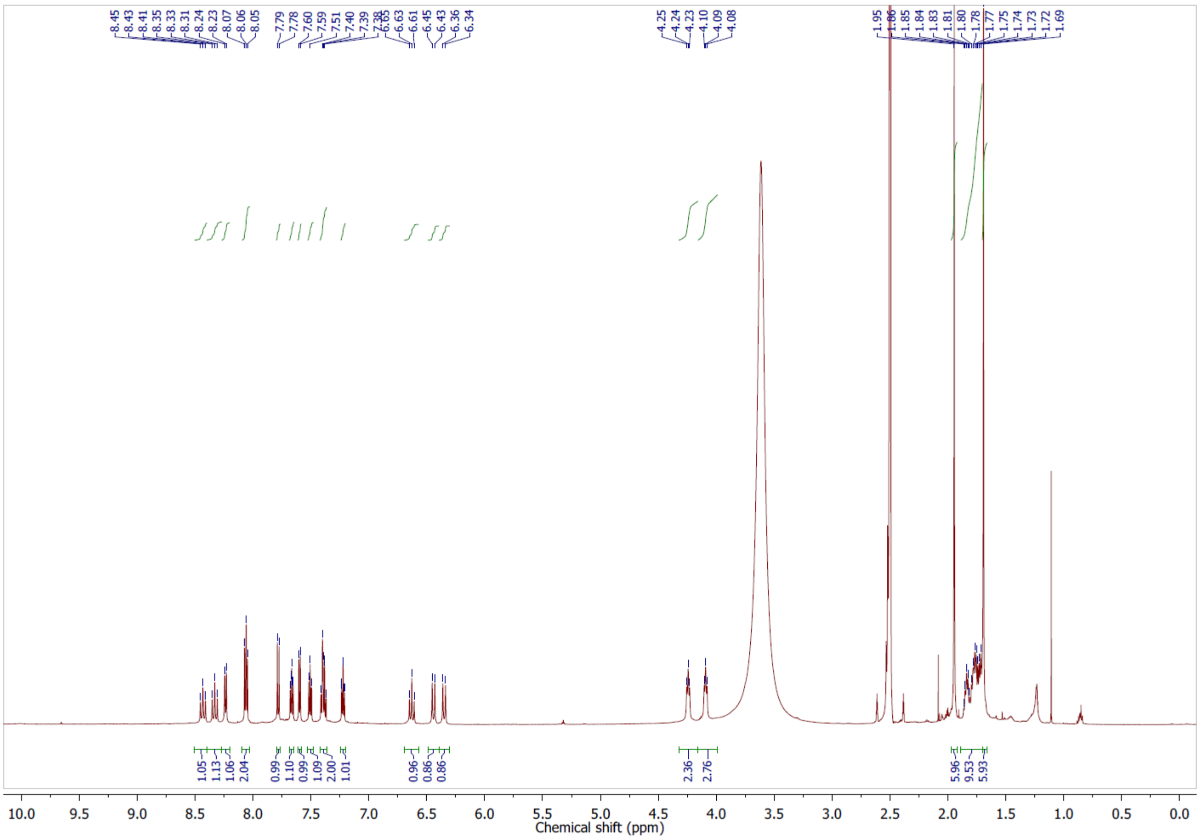

Figure S15. ^{1}H-NMR spectrum of dye **8**.

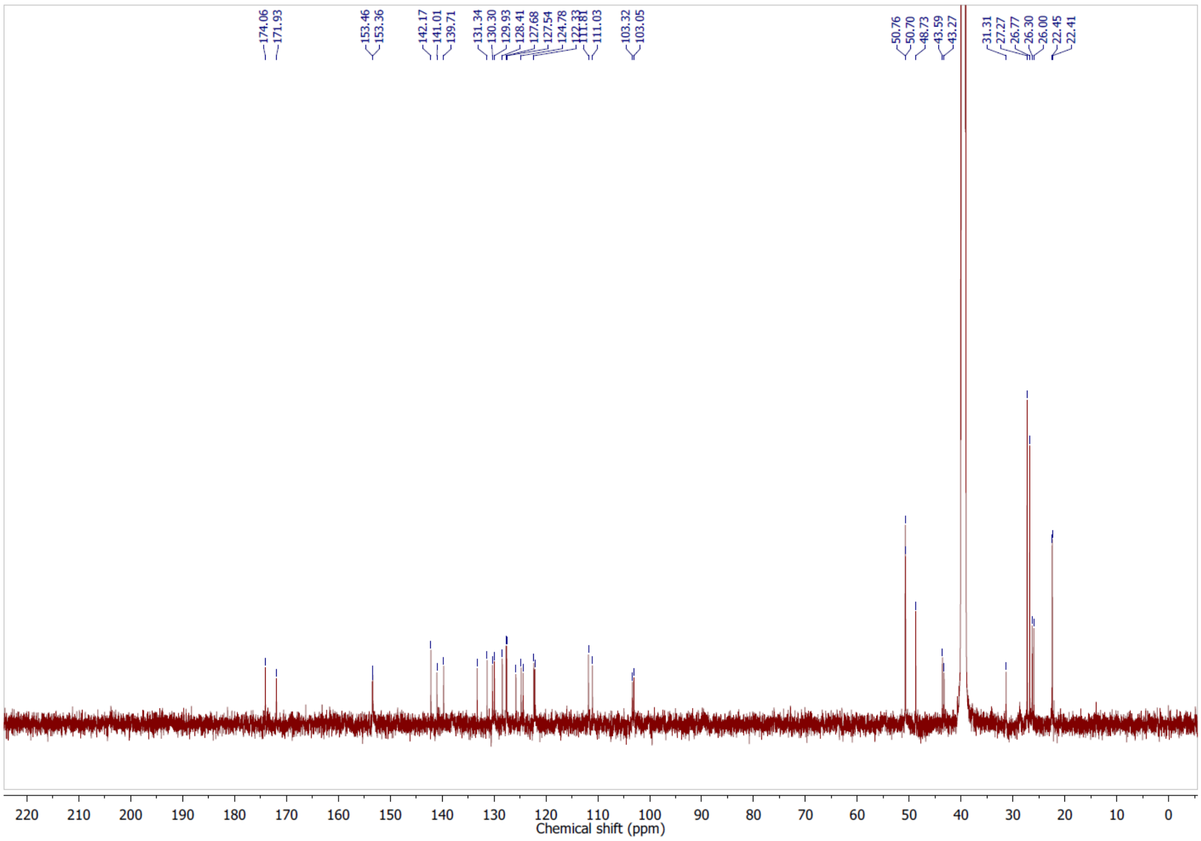

Figure S16. ^{13}C-NMR spectrum of dye **8**.

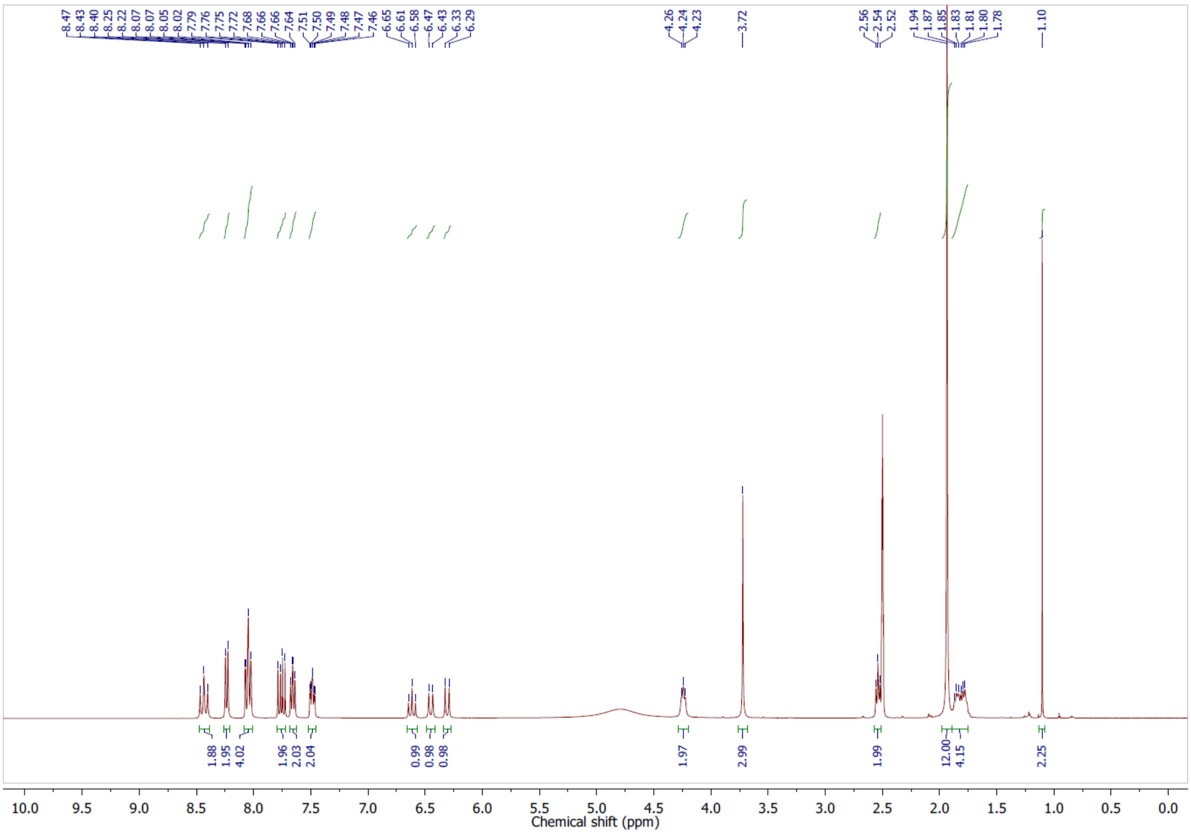

Figure S17.^{1}H-NMR spectrum of dye **9**.

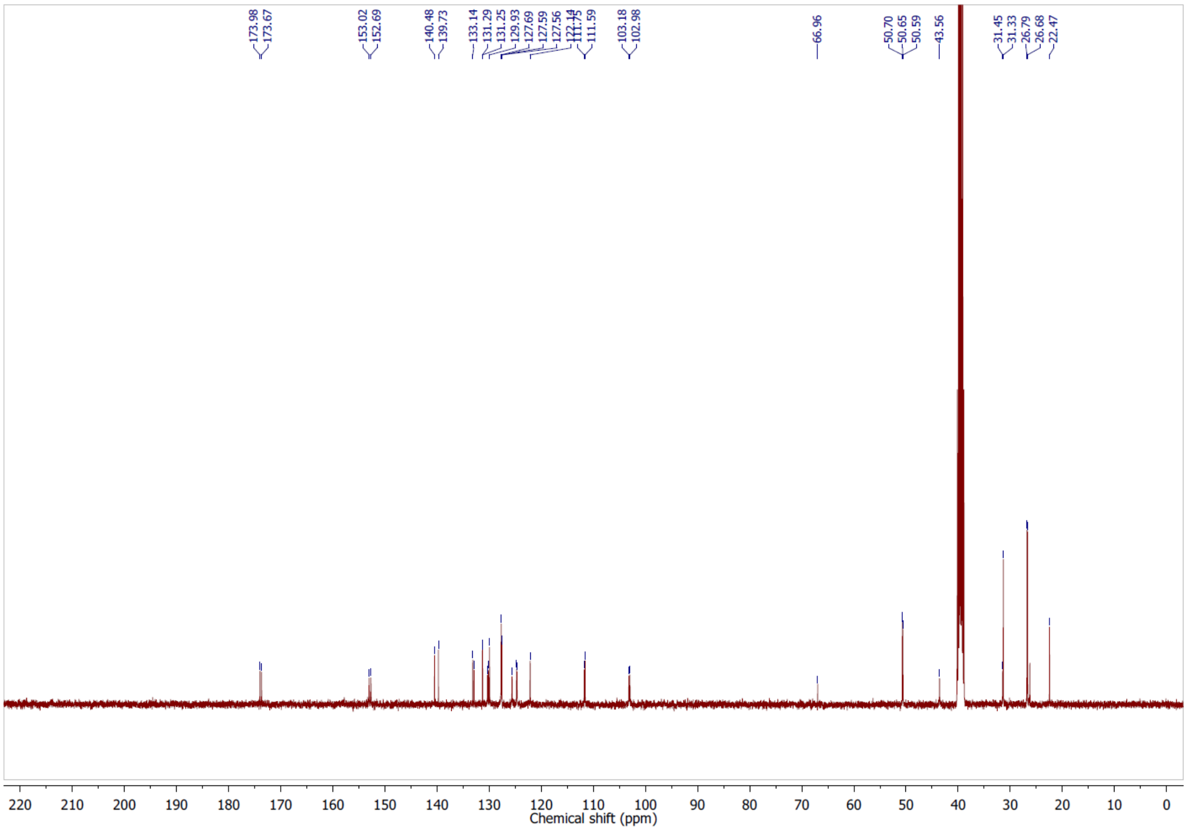

Figure S18. ^{13}C-NMR spectrum of dye **9**.

Mass analysis of cyanine dyes **1** - **9**

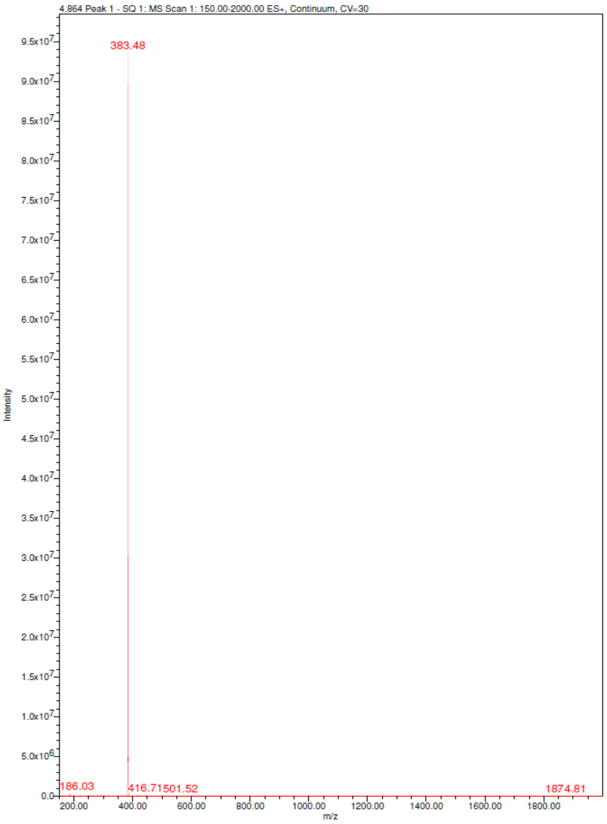

Figure S19. Mass analysis of **1**.

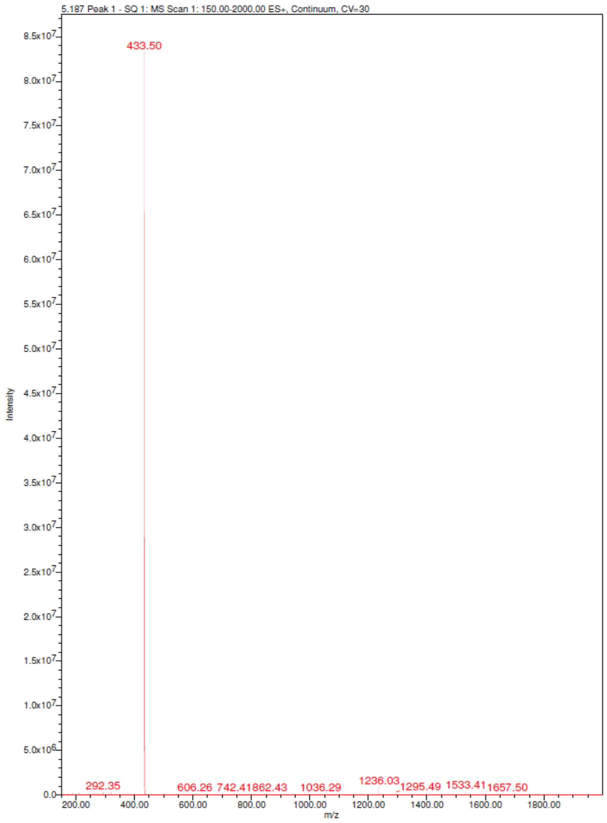

Figure S20. Mass analysis of **2**.

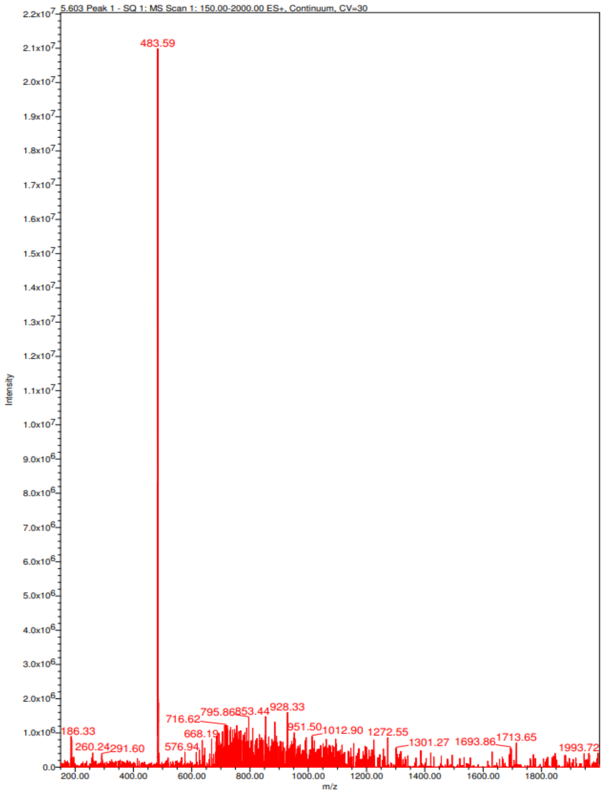

Figure S21. Mass analysis of **3**.

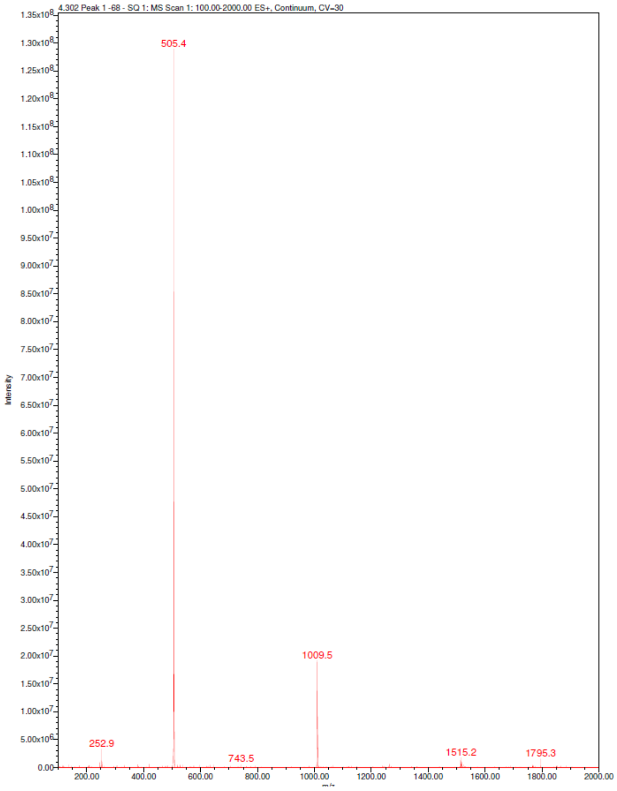

Figure S22. Mass analysis of **4**.

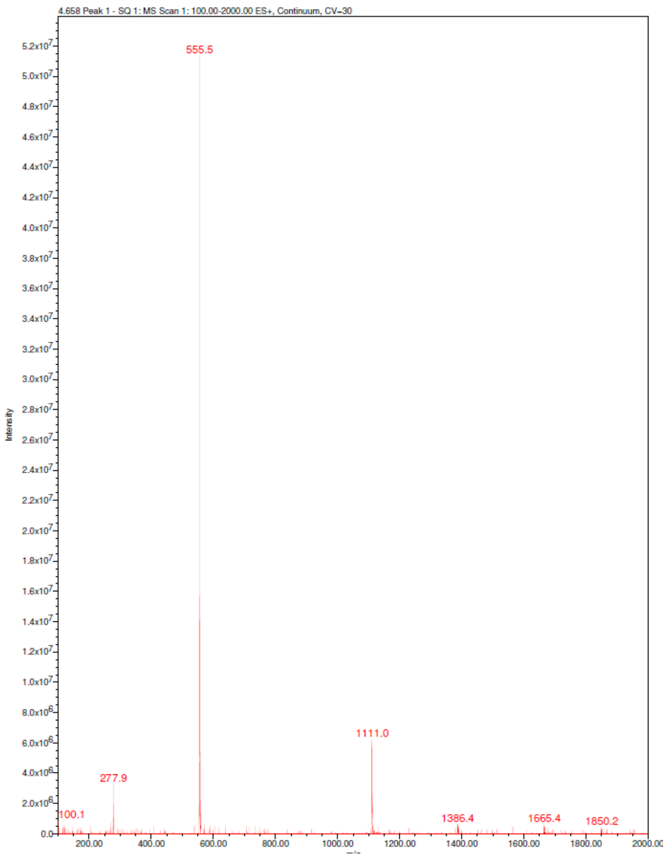

Figure S23. Mass analysis of **5**.

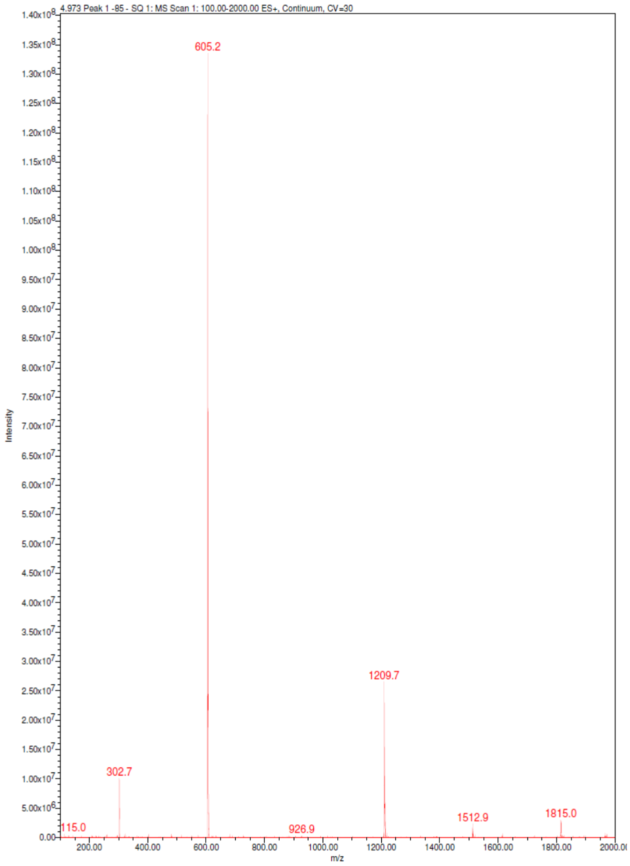

Figure S24. Mass analysis of **6**.

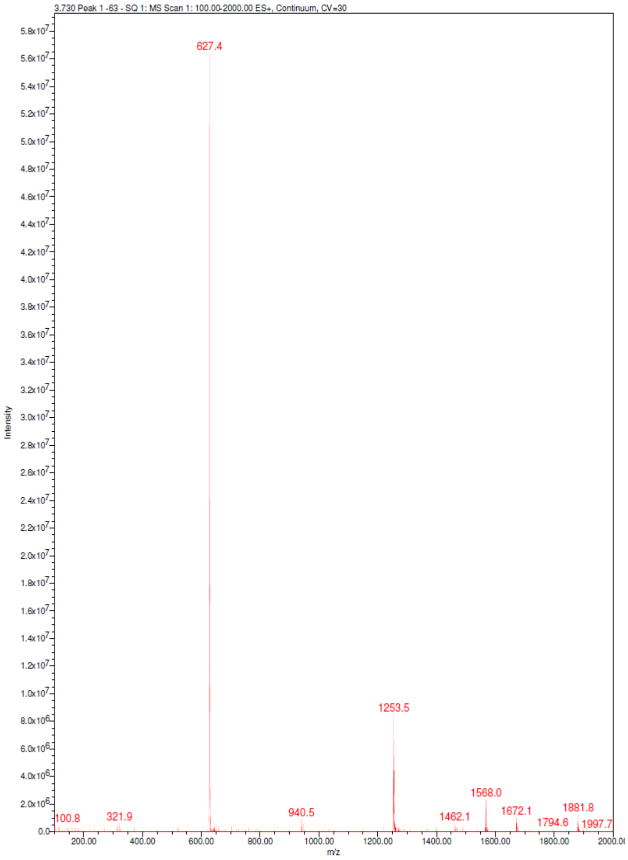

Figure S25. Mass analysis of **7**.

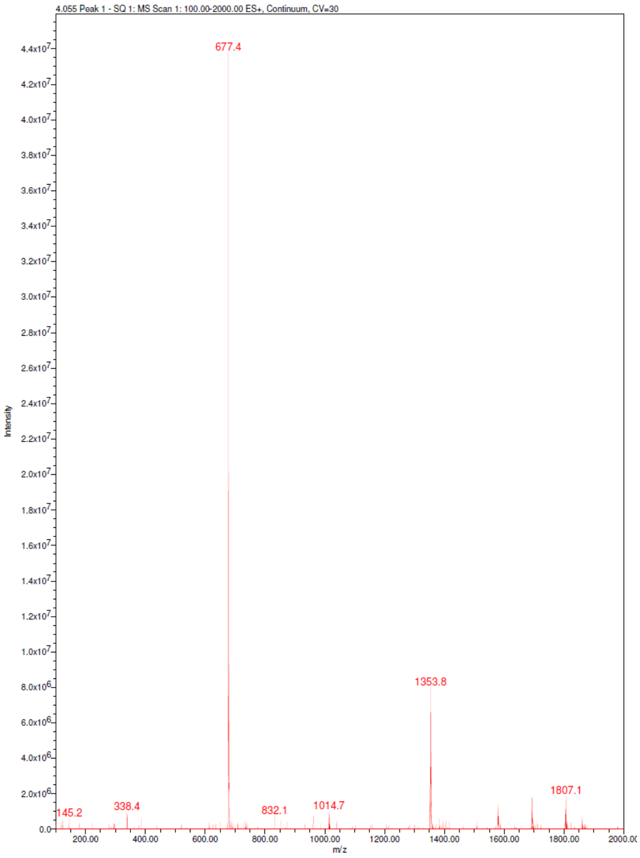

Figure S26. Mass analysis of **8**.

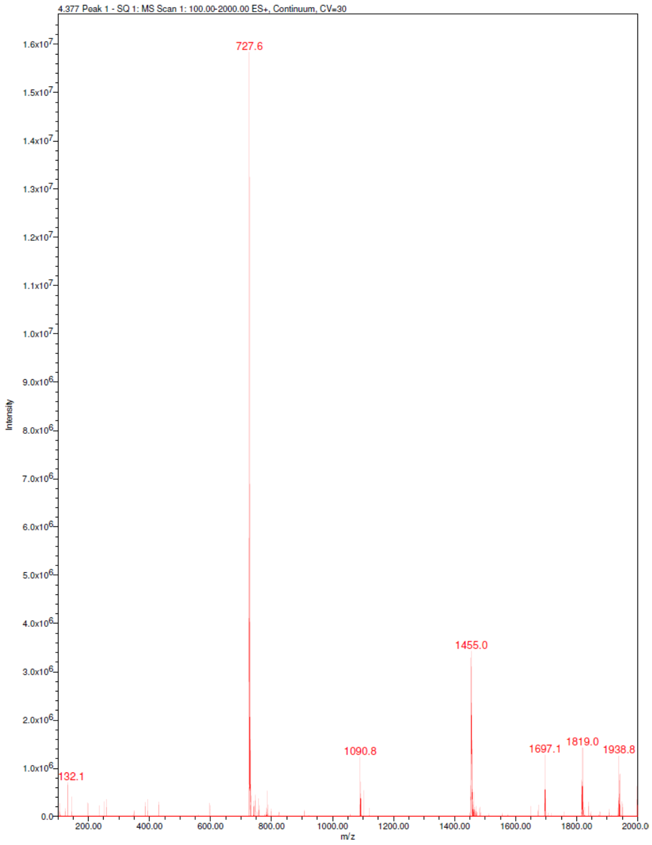

Figure S27. Mass analysis of **9**.

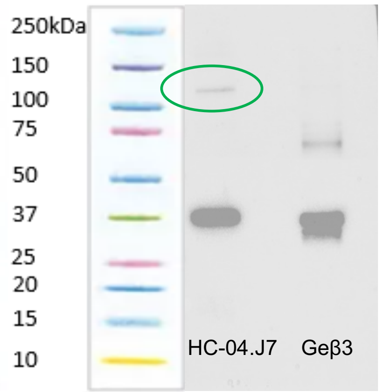

Figure S28. Assessment of the presence of liver-specific influx transporters OATP1B1 in hepatocytes HC-04.J7 and control epithelial cells Geβ3. OATP1B1 specific band for the HC-04.J7 circled in green [1]. A band at approximately 72 kDa for Geβ3 represents unglycosylated form of OATP1B1 [2].

Table S1. Chemical and optical properties of ICG.

	**ICG**
Abs_{max}/Em_{max} (pH5), nm	779/817
Abs_{max}/Em_{max} (pH8), nm	779/817
Brightness*, $cm^{-1}M^{-1}$	1 672
LogP	-0.18± 0.04
Serum binding, %	70

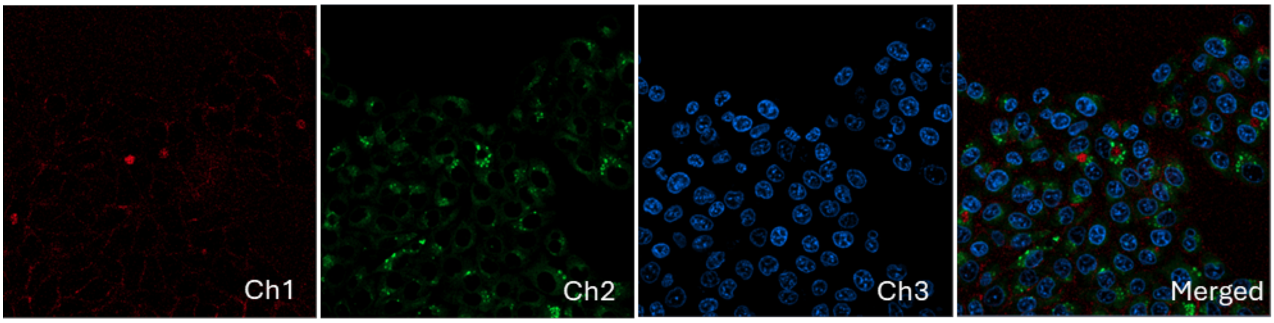

Figure S29. Confocal evaluation of the ICG in the HC-04.J7 cell line. Ch1- ICG signal (in red), Ch2 - lysosomal staining (in green), Ch3- nuclei (in blue).

References

1. Schweikhard, E.S., et al., *Role of N-glycosylation in renal betaine transport.* Biochem J, 2015. **470**(2): p. 169–79.
2. Chapy, H., et al., *PBPK modeling of irbesartan: incorporation of hepatic uptake.* Biopharm Drug Dispos, 2015. **36**(8): p. 491–506.
